# Accumulation of senescent cells in the adrenal gland induces hypersecretion of corticosterone via IL1β secretion

**DOI:** 10.1111/acel.14206

**Published:** 2024-05-20

**Authors:** Noriyuki Okudaira, Mi‐Ho Akimoto, Takao Susa, Miho Akimoto, Harumi Hisaki, Masayoshi Iizuka, Hiroko Okinaga, Julio A. Almunia, Noboru Ogiso, Tomoki Okazaki, Mimi Tamamori‐Adachi

**Affiliations:** ^1^ Department of Biochemistry Teikyo University School of Medicine Tokyo Japan; ^2^ Medical Education Centre Teikyo University School of Medicine Tokyo Japan; ^3^ Department of Internal Medicine Teikyo University School of Medicine Tokyo Japan; ^4^ Department of Laboratory of Experimental Animals National Center for Geriatrics and Gerontology (NCGG) Obu Aichi Japan

**Keywords:** adrenal gland, glucocorticoids, IL1β, p16^INK4A^, SF1 (Ad4BP/SF1)

## Abstract

Aging progresses through the interaction of metabolic processes, including changes in the immune and endocrine systems. Glucocorticoids (GCs), which are regulated by the hypothalamic–pituitary–adrenal (HPA) axis, play an important role in regulating metabolism and immune responses. However, the age‐related changes in the secretion mechanisms of GCs remain elusive. Here, we found that corticosterone (CORT) secretion follows a circadian rhythm in young mice, whereas it oversecreted throughout the day in aged mice >18 months old, resulting in the disappearance of diurnal variation. Furthermore, senescent cells progressively accumulated in the zF of the adrenal gland as mice aged beyond 18 months. This accumulation was accompanied by an increase in the number of Ad4BP/SF1 (SF1), a key transcription factor, strongly expressing cells (SF1‐high positive: HP). Removal of senescent cells with senolytics, dasatinib, and quercetin resulted in the reduction of the number of SF1‐HP cells and recovery of CORT diurnal oscillation in 24‐month‐old mice. Similarly, administration of a neutralizing antibody against IL1β, which was found to be strongly expressed in the adrenocortical cells of the zF, resulted in a marked decrease in SF1‐HP cells and restoration of the CORT circadian rhythm. Our findings suggest that the disappearance of CORT diurnal oscillation is a characteristic of aging individuals and is caused by the secretion of IL1β, one of the SASPs, from senescent cells that accumulate in the zF of the adrenal cortex. These findings provide a novel insight into aging. Age‐related hypersecretory GCs could be a potential therapeutic target for aging‐related diseases.

## Graphical abstract

Corticosterone is highly secreted throughout the day in older male mice, resulting in the loss of diurnal variation. Senolytics improve CORT levels along with the removal of senescent cells accumulated in the adrenal gland. IL1β expression is increased in aged adrenals, and causes overproducing CORT as well as inducing adrenocortical cells to be a senescent state.

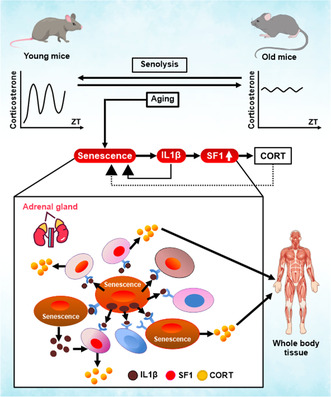

**Abbreviations**
53BP1: p53 binding protein 1
AC: adrenal cortex
ACTH: adrenocorticotropic hormone
AM: adrenal medulla
CORT: corticosterone
CYP11A1: cytochrome P450 family 11 subfamily A member 1
CYP11B1: cytochrome P450 family 11 subfamily B member 1
Dex: dexamethasone
GC: glucocorticoid
GR: glucocorticoid receptor
HPA: Hypothalamus‐Pituitary‐Adrenal axis
IL1β: interleukin 1 beta
NF‐κB: nuclear factor‐κB
p16: p16^INK4a^
pRB: retinoblastoma protein
SASP: senescence‐associated secretory phenotype
SA‐β‐gal: senescence‐associated β‐galactosidase
SF1: Ad4BP/SF1
StAR: steroidogenic acute regulatory protein
zF: zona fasciculata
zG: zona glomerulosa
zR: zona reticularis
ZT: zeitgeber time
γH2AX: phospho‐histone H2A family member X

## 1 INTRODUCTION

Aging is a major risk factor for dysfunction and disease development in humans. Recent advances in aging research have identified the *p53‐p21* ^(WAF1/CIP1)^ and *p16* ^*Ink4a*^ (*p16*) retinoblastoma protein (*pRB*) pathways as important pathways involved in senescence induction (Beauséjour et al., 2003); (Sharpless & DePinho, 2005). As for the causes of cellular senescence in humans, telomere length shortening, oncogene activation, and oxidative stress are induced by a number of physiological and pathological factors (Herbig & Sedivy, 2006) (Jeyapalan & Sedivy, 2008). Moreover, senescent cells secrete inflammatory cytokines, chemokines, and growth factors, which is known as the senescence‐associated secretory phenotype (SASP) (Rodier & Campisi, 2011). In recent years, the activation of long noncoding RNAs and transposons has been associated with aging, and antiaging drugs targeted to these are being researched (Akimoto et al., 2022); (De Cecco et al., 2019); (Lozano‐Vidal et al., 2019); (Okudaira et al., 2022). Previously, in mice monitored for p16 expression, the accumulation of senescent cells resulted in tissue damage and senile disease (Yamakoshi et al., 2009). To determine the role of senescence in aging‐related diseases, the INK‐ATTAC mouse model was established in which p16‐expressing senescent cells could be eliminated (Baker et al., 2011). More recently, several senolytics, drugs that target cellular senescence, have been tested. These studies have reported that the removal of senescent cells improved functional impairment in individuals with aging. Therapeutics that target senescent cells may prevent aging comorbidities by targeting a fundamental aging mechanism (Baker et al., 2016); (Johmura et al., 2021); (Ogrodnik et al., 2019).

The adrenal gland is an endocrine hormone‐producing organ essential for homeostasis. The cortex of the adrenal gland is divided into layers: the zona glomerulosa (zG), zona fasciculata (zF), and zona reticularis (zR), each of which produces different steroid hormones (Pignatti & Flück, 2021). Glucocorticoid (GC), which is produced by the zF of the adrenal gland, regulates metabolism, development, and immune responses via the glucocorticoid receptor (GR). The adrenal gland is a key component of the hypothalamus–pituitary–adrenal (HPA) axis, which plays an important role in the adaptation of organisms to various stressors. Adrenocorticotropic hormone (ACTH) binds to the melanocortin 2 receptor (MC2R), which is located in the adrenal cortical fasciculate layer, another core player of the HPA axis. It activates adenylyl cyclase, resulting in cAMP production followed by PKA activation. Subsequent activation of specific transcription factors, such as Ad4BP/SF1 (SF1), promotes the expression of factors in the adrenocortical steroidogenic pathway, steroidogenic acute regulatory protein (StAR), cytochrome P450c11 (encoded by CYP11A1), and cytochrome P45011B1 (encoded by CYP11B1) at the transcriptional level, resulting in GC production (Schimmer et al., 2011); (Shih et al., 2008); (Suda et al., 2011). SF1 is a nuclear receptor transcription factor essential for adrenal function and formation and is involved in steroid hormone production. SF1‐binding regions have been identified in the promoters of StAR, CYP11A1, and CYP11B1 (Honda et al., 1993); (Morohashi et al., 1993). The GC precursor cholesterol cannot permeate the mitochondrial membrane and requires StAR to be transported from the cytosol into the mitochondria. CYP11A1 and CYP11B1 are enzymes required for the conversion of cholesterol to GC (Harvey, 2016). GC is a diurnally varying hormone that plays an important role in homeostatic mechanisms such as sleep as well as in gluconeogenesis, anti‐inflammation (Ikeda et al., 2013), cortical plasticity (Dufour et al., 2022), anxiety‐like behavior (Panagiotidou et al., 2014), the diurnal rhythm of neural stem/precursor cells (Schouten et al., 2020), and proliferation in the dentate gyrus (Schouten et al., 2020).

Previous reports showed that human plasma GC increases with age (Van Cauter et al., 2000) and that the diurnal rhythm is irregular (Ferrari et al., 2008); (Yiallouris et al., 2019). The plasma level of mouse corticosterone (CORT), a hormone equivalent to human GC and whose diurnal rhythm allows young mice to adapt to stressful environments (Gong et al., 2015); (Son et al., 2008), has also been reported to increase with age (Ferrari et al., 2008); (Schouten et al., 2020). We previously reported that a human adrenal‐derived cell line (H295R) with DNA damage exhibits enhanced GC production mediated by GADD45A, which is a stress factor that induces cellular senescence (Tamamori‐Adachi et al., 2018). When the regulation of GC secretion is disrupted, the body is unable to respond to stress (Lightman et al., 2020). Excess plasma GC results in the development of diseases, such as osteoporosis, type II diabetes mellitus, hyperlipidemia, and immune system disorders, which are also diseases that are more prevalent with age. In fact, GC has been reported to be implicated in the cause of aging, resulting from reactive oxygen species production, mitochondrial dysfunction, and endoplasmic reticulum stress (Chrousos, 1995); (Gassen et al., 2017); (Sapolsky et al., 2000). However, limited evidence suggests that there is an age‐related GC production mechanism that affects aging itself and the development of age‐related diseases. This study aimed to compare CORT production between young and old mice and analyze the molecular and cellular mechanisms of age‐related changes in the regulation of GC secretion in the adrenal gland.

## 2 RESULTS

### 2.1 GC is highly secreted throughout the day in older male mice, resulting in diurnal variation loss

**FIGURE 1:**
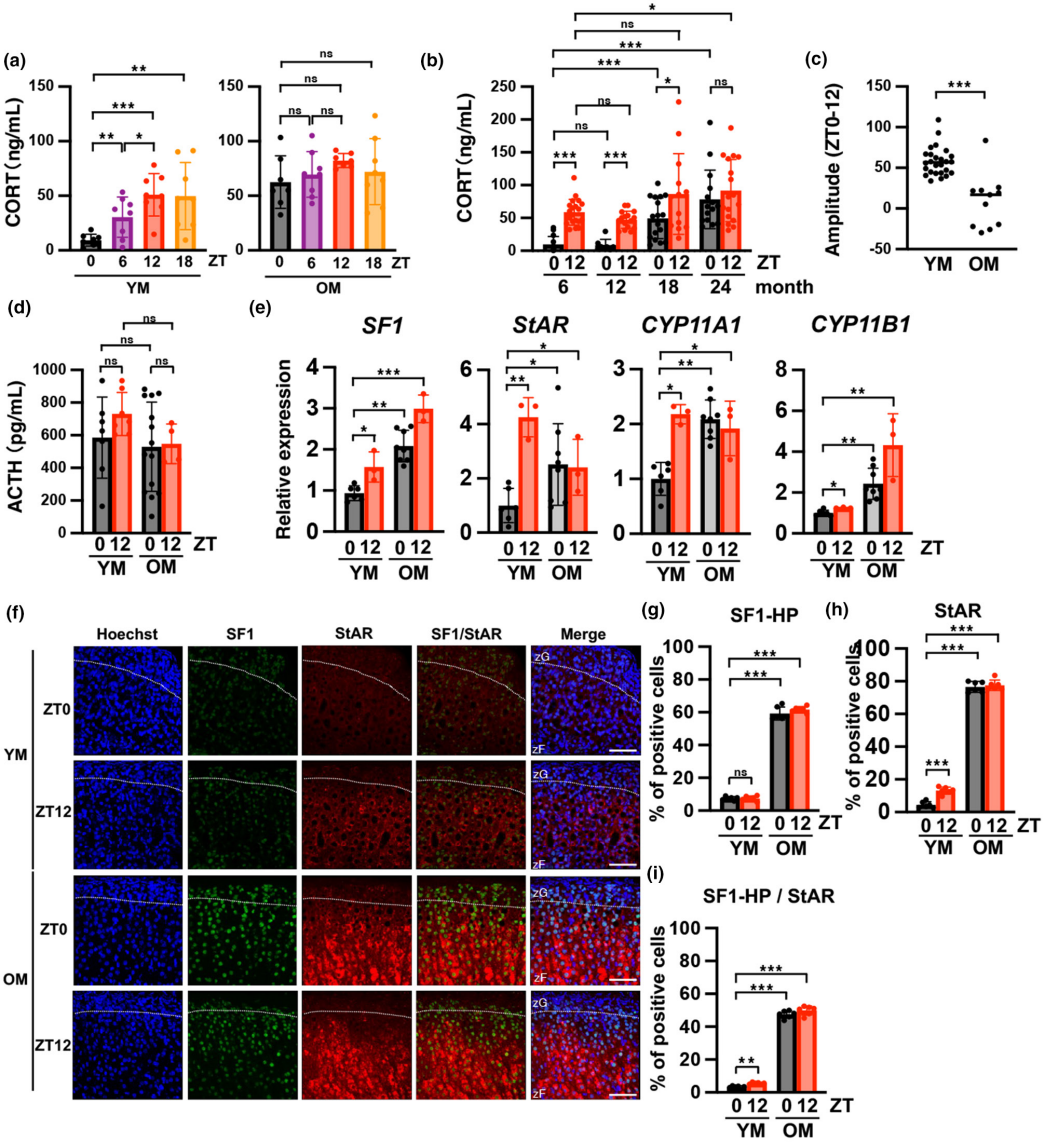
Corticosterone synthesis increases throughout the day with age with disappearance of diurnal variation in male mice. (a) Comparison of CORT levels in mouse plasma samples collected every 6 h (ZT0, 6, 12, 18; 08:00, 14:00, 20:00, 2:00, lights on: ZT0, lights off: ZT12) in 6‐month‐old mice (YM) and 24‐month‐old mice (OM) in the diurnal rhythm. (b) CORT production increased at ZT0 and ZT12 in mice as aging progressed. CORT levels were measured in plasma samples using ELISA. (c) The difference between ZT0 and ZT12 CORT levels in the same individual was quantified. For the 6 M sample (YM), *n* = 26. For the 24 M sample (OM), *n* = 11. (d) Comparison of ACTH concentrations between YM and OM at ZT0 and ZT12. ACTH levels were measured in plasma samples using ELISA. (e) Analysis of the mRNA expression of genes encoding steroid‐producing enzymes in YM and OM at ZT0 and ZT12. All data are presented as relative values and were normalized to the expression of TATA‐binding protein (TBP). (f) Immunofluorescent images of SF1 and StAR using a confocal laser scanning microscope in the adrenal gland in YM and OM at ZT0 and ZT12, respectively. Scale bar (solid line) = 50 μm. zF, zona fasciculata; zG, zona glomerulosa. (g and h) The percentage of SF1‐HP and StAR‐positive cells was quantified in YM and OM at ZT0 and ZT12. (i) The percentage of SF1‐HP positive cells among StAR‐positive cells in zF was quantified in YM and OM at ZT0 and ZT12. SF1‐HP; SF1‐high positive cells. Data are shown as the mean ± SD. Asterisks indicate statistical significance (*p < 0.05, **p < 0.001, ***p < 0.0001, ns; not significant). The quantitative results of immunostaining are summarized in Table S1.

To confirm the diurnal rhythm of CORT, plasma samples were collected every 6 h (ZT0, 6, 12, 18; 08:00, 14:00, 20:00, 2:00, lights on: ZT0, lights off: ZT12) from 6‐month‐old (young mice: YM) and 24‐month‐old (older mice: OM) mice. CORT concentrations were measured in plasma samples using enzyme‐linked immunosorbent assay (ELISA; Figure 1a). Male YM exhibited a diurnal rhythm, with a bottom value of <10 ng/mL at ZT0 and a peak value at ZT12. For OM, higher values than the YM peak were obtained at all time points (Figure 1a). By contrast, in female mice, lowest and highest CORT concentrations were measured at ZT0 and ZT12, respectively, not only in YM, but also in OM, indicating that diurnal variation was maintained in OM (Figure S1). Therefore, we focused on changes in CORT concentrations in male mice with aging. CORT AM (ZT0) levels were significantly increased in 18‐month (18 M) and 24‐month (24 M)‐old mice in an age‐dependent manner compared with 6‐month (6 M) and 12‐month (12 M)‐old mice (Figure 1b). CORT PM (ZT12) levels were stable at 6 M and 12 M and were upregulated at 18 M and 24 M. The differences between ZT0 and ZT12 CORT concentrations for each individual as diurnal amplitudes were determined, and a significant reduction was observed in OM compared with YM (Figure 1c). Since GC secretion is known to be mainly regulated by the HPA axis (Harvey, 2016), we assayed ACTH, which is upstream of CORT in the HPA axis, to investigate the involvement of the HPA axis in CORT hypersecretion in ZT0. As shown in Figure 1d, the mean plasma ACTH level in YM was only slightly higher at ZT12 than at ZT0, whereas in OM, it was similar at ZT0 and ZT12 and was the same as that at ZT0 in YM. Taken together, these findings indicate that the increase in ZT0 CORT levels in OM was not accompanied by an increase in ACTH levels, suggesting that it is independent of the HPA axis.

CORT is produced in the zona fasciculata (zF) of the adrenal gland (Pignatti & Flück, 2021). To analyze expression of aging and senescence, and steroid‐producing gene groups, the zF regions of the adrenal gland, which are CORT‐producing tissues, were isolated using laser microdissection (LMD), followed by RNA extraction. Sampling times were the ZT0 (morning) and ZT12 (evening) points in YM and OM to determine the mechanism underlying the increase in CORT production in OM. The mRNA expression of *SF1*, a master transcription factor that regulates the expression of almost all genes involved in steroid hormone synthesis, was increased 2.1‐fold in OM (ZT0) compared with YM (ZT0) (Figure 1e). Furthermore, the mRNA expression levels of *StAR*, *CYP11A1*, and *CYP11B1* were increased more than twofold in OM (ZT0) compared with YM (ZT0) (Figure 1e). Immunostaining of the adrenal glands was performed using SF1 and StAR antibodies (Figure 1f, Figure S2a). SF1 was expressed in both zG and zF of the adrenal gland, whereas StAR was strongly expressed in zF (Figure 1f). The percentages of SF1‐highly positive cells were approximately 10% (ZT0 and ZT12, YM), and around 60% (ZT0 and ZT12, OM), which indicate an approximate 6‐fold increase in zF of OM (Figure 1g). The criterion for SF1‐highly positive (HP) cells was determined as cells with intensity greater than “9” as shown in Figure S2b, while the intensity of most cells in the young mice was less than nine. The percentages of StAR‐positive cells were approximately 4.5% (YM ZT0), 13% (YM ZT12), and 80% (ZT0 and ZT12, OM) (Figure 1h). Thus, the rate of StAR‐positive cells was significantly higher at ZT12 than at ZT0 in YM, which was consistent with the mRNA expression results. Furthermore, at ZT0, the number of these cells was about 17‐fold larger in OM than in YM. The percentage of cells expressing both high‐level‐SF1 and StAR was less than 10% (ZT0 and ZT12, YM), and more than 45% (ZT0 and ZT12, OM) (Figure 1i). These results suggest that the age‐dependent increase in plasma CORT levels at ZT0 correlates with the upregulation of SF1 and StAR expression.

### 2.2 Senescent cells progressively accumulate in the adrenal glands with aging

**FIGURE 2:**
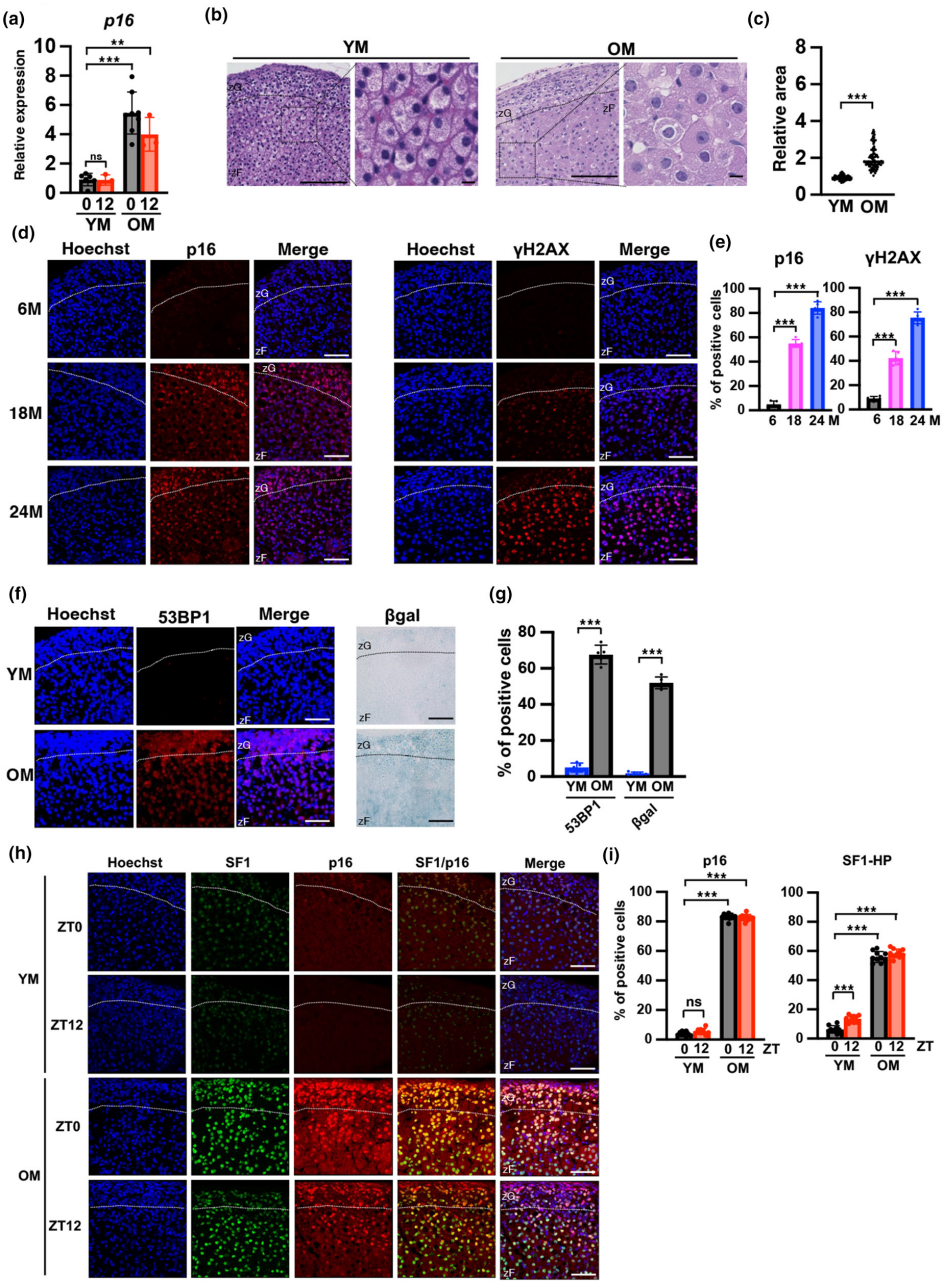
Accumulation of cells expressing senescence markers in the zF layers of adrenal glands of old mice. (a) Analysis of mRNA expression of the p16 senescence marker in YM and OM at ZT0 and ZT12. Data are presented as relative values and were normalized to TBP. (b) Tissue sections prepared from YM and OM adrenal glands were stained with hematoxylin–eosin (H&E) for histological evaluation and assessed via light microscopy. (c) The size of cells in the H&E‐stained adrenal glands was quantified in YM and OM. (d) Immunofluorescent images of p16 and γH2AX in the adrenal gland at 6, 18, and 24 M. Each section was stained with an anti‐p16 and γH2AX antibody and observed using a confocal laser scanning microscope. (e) The number of p16‐ and γH2AX‐positive cells in zF was quantified. (f) Immunofluorescent images of 53BP1 and β‐galactosidase (βgal) in the adrenal gland of YM and OM. Each section was stained with an anti‐53BP1 antibody and for β‐galactosidase activity using the X‐gal substrate and observed using a confocal laser scanning microscope or a light microscope, respectively. (g) Quantification of 53BP1‐ and βgal‐positive cells. (h) Immunofluorescent images of p16 and SF1 using a confocal laser scanning microscope in the adrenal gland of YM and OM at ZT0 and ZT12. (i) The percentage of p16‐ and SF1‐HP cells in zF was determined in YM and OM at ZT0 and ZT12. Scale bar (solid line) = 50 μm. zF, zona fasciculata; zG, zona glomerulosa. Data are shown as the mean ± SD. Asterisks indicate statistical significance (**p < 0.001, ***p < 0.0001, ns; not significant). The quantitative results of immunostaining are summarized in Table S1.

To determine whether adrenal glands also exhibited the aging phenotype in OM, we evaluated the characteristics of senescent cells. Quantification of *p16* mRNA expression in the zF of adrenal gland showed an increase in OM (Figure 2a). Hematoxylin–eosin (H&E) staining of YM and OM adrenal glands revealed that the zF cells were aligned in YM, whereas cell alignment was disrupted in OM (Figure 2b). Quantitative analysis of cell size indicated that the cells were significantly larger in OM than in YM (Figure 2c). H&E staining of female adrenal tissue was also similar to that of males (Figure S3). To further confirm whether senescent cells accumulated in the adrenal glands in OM, immunofluorescence of the cellular senescence markers p16 and γH2AX was performed. As shown in Figure 2d,e, the percentage of p16 positive cells in zF was very low in 6 M‐old mice, increased modestly in 18 M, and increased markedly to 80% in 24 M. The percentage of γH2AX‐positive cells was also very similar to that of p16 (Figure 2d,e, Figure S4a,b). In addition, we assessed the expression of 53BP1 and β‐galactosidase as other senescence markers. The percentage of cells positive for 53BP1 increased by approximately 14‐fold (53BP1:5.0% in YM and 68% in OM) and the positive area for β‐galactosidase was approximately 2% in YM and 50% in OM (Figure 2f,g). The results indicate strong expression of senescence markers in the zF regions, thus confirming the accumulation of senescent cells in the zF of adrenal glands of OM. Because SF1 is a master regulator of adrenal gland development and steroid hormone production, SF1‐HP cells are considered to be CORT hypersecreting cells in OM. Costaining of p16 and SF1 in 6 M, 18 M, and 24 M mouse adrenal glands at ZT0 was performed to determine association of enhanced CORT secretion with the accumulation of senescent cells in the adrenal glands of OM. As shown in Figure S4c–e, the number of SF1‐HP cells increased in an age‐dependent manner, correlating with p16‐positive cells in the zF region, indicating that senescent cell accumulation is involved in CORT hypersecretion (Figure 2h,i, Figure S4f).

### 2.3 Senolytic drugs improve CORT levels along with the removal of senescent cells in the adrenal gland

**FIGURE 3:**
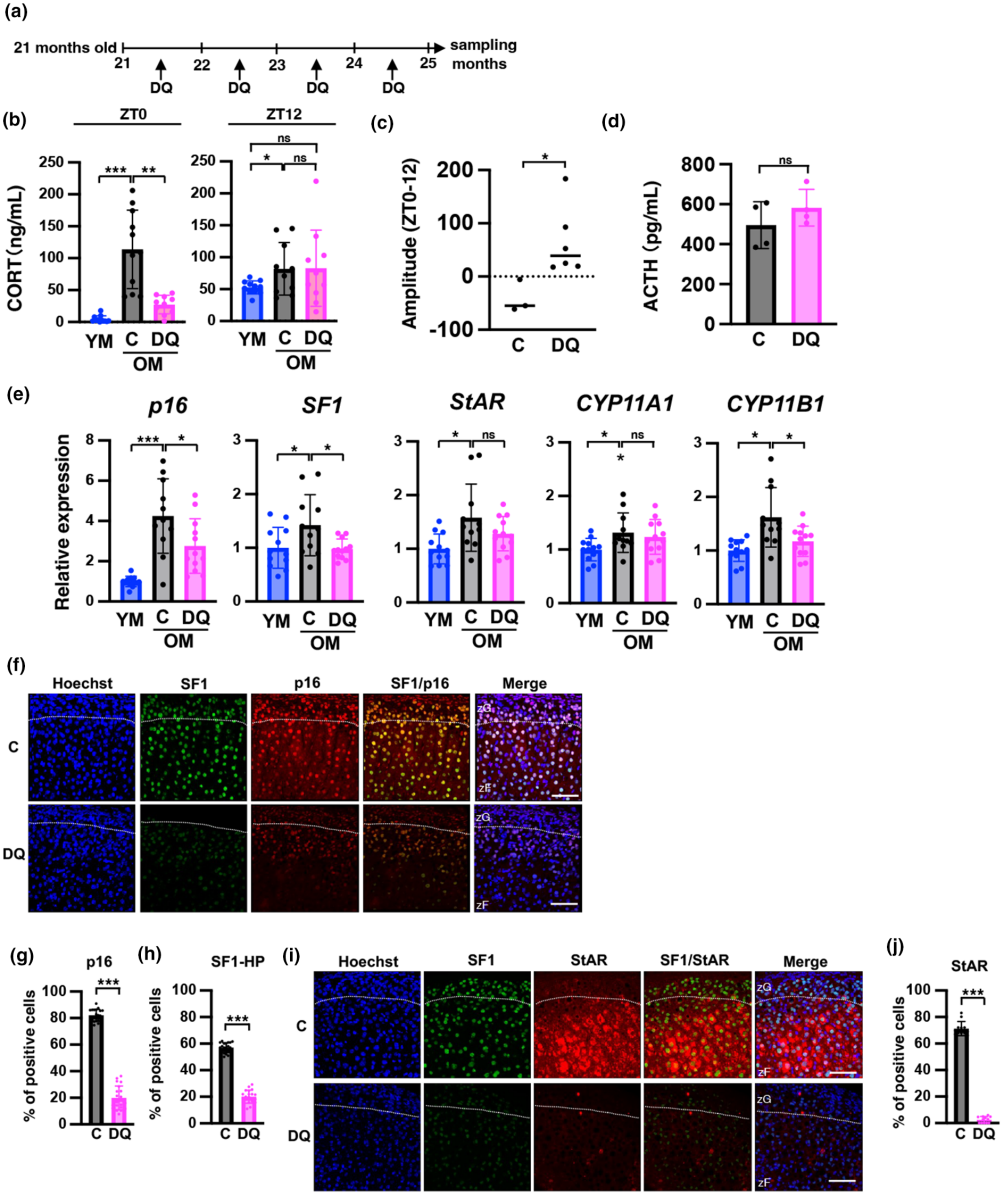
Senolytic drugs suppress the production of CORT and the expression of SF1 at ZT0 in old mice. (a) Schedule of DQ administration experiments. (b) CORT levels at ZT0 of YM and OM treated with senolytic drugs were determined (C: control, DQ: senolysis). (c) The amplitude of ZT0 and ZT12 in CORT was recovered by the senolytic drug treatment of OM. The difference between ZT0 and ZT12 CORT levels in the same individual was quantified. For the control sample (C), *n* = 3. For senolytic sample (DQ), *n* = 6. (d) Comparison of ACTH concentrations between control (C) and senolytic (DQ) treatment of OM. (e) Analysis of the mRNA expression of genes encoding senescence markers and steroid‐producing enzymes in YM and DQ‐treated mice. All data are presented as relative values and were normalized to TBP. (f and i) Immunofluorescent images of p16, SF1, and StAR in the adrenal gland of DQ‐treated mice. Each section was stained with an anti‐p16, SF1, and StAR antibody and observed using a confocal laser scanning microscope. Scale bar (solid line) = 50 μm. zF, zona fasciculata; zG, zona glomerulosa. (C: control, DQ: senolysis). (g, h, and j) The number of p16‐, SF1‐, StAR‐positive cells in zF was quantified. SF1‐HP; SF1‐high positive cells. Data are shown as the mean ± SD. Asterisks indicate statistical significance (*p < 0.05, **p < 0.001, ***p < 0.0001, ns; not significant). The quantitative results of immunostaining are summarized in Table S1.

To determine whether senescent cells are involved in CORT overproduction, we administered dasatinib (D) and quercetin (Q)—senolytic drugs known to remove senescent cells (Farr et al., 2017)—to OM and analyzed CORT levels, senescence markers, and steroid‐related factors. The experimental schedule is shown in Figure 3a. Immunostaining of liver tissue was performed to determine whether senescent cells in the liver were eliminated by DQ treatment. The results showed that DQ markedly eliminated senescent cells in the liver, as confirmed by p16, γH2AX, and β‐galactosidase expression (Figure S5a). In the adrenal glands, the number of cells expressing the aging markers p16, γH2AX, or 53BP1 was significantly reduced by DQ, respectively, as evidenced by the staining area of β‐galactosidase (Figure S5b). The liver and adrenal gland results confirmed that DQ treatment eliminates senescent cells. Next, we determined whether DQ suppresses the increase in blood CORT levels at ZT0 in OM. The results indicated that CORT levels at ZT0 improved in the OM of the DQ group but not at ZT12 (Figure 3b). Calculation of the individual amplitude of CORT between ZT0 and ZT12 indicated that the DQ‐treated group significantly recovered (Figure 3c). In contrast, there were no differences in plasma ACTH concentrations at ZT0 between the control and DQ groups (Figure 3d). We measured the expression levels of aging‐related and steroid‐producing genes at ZT0. The expression of *p16*, *SF1*, and *CYP11B1* was significantly decreased in the DQ‐treated group (Figure 3e). *StAR* and *CYP11A1* expression was downregulated by DQ but not significantly (StAR and CYP11A1; OM control vs. OM DQ, *p* = 0.158, *p* = 0.599, respectively). Immunostaining analysis using samples at ZT0 revealed that the number of SF1‐HP cells was significantly decreased by DQ treatment as well as p16‐positive cells (Figure 3f–h, Figure S5c). Further analysis of StAR by double‐staining with SF1 revealed that DQ treatment significantly reduced StAR expression to almost basal levels (Figure 3i,j, Figure S5d); however, this result was not consistent with the results of gene expression analysis using RT‐qPCR (Figure 3e,i,j), which indicates that StAR may be regulated not only by transcription but also through post‐transcriptional mechanisms. These results indicate that senolytic drugs eliminate senescent cells in the zF and improve the level and diurnal oscillations of CORT accompanied by decreased expression of steroid‐producing factors. Excess secretion of CORT in OM may result from an increase in senescent cells in the zF region of the adrenal gland.

### 2.4 IL1β expression is increased in aged adrenals, but not accompanied by increased macrophage infiltration

**FIGURE 4:**
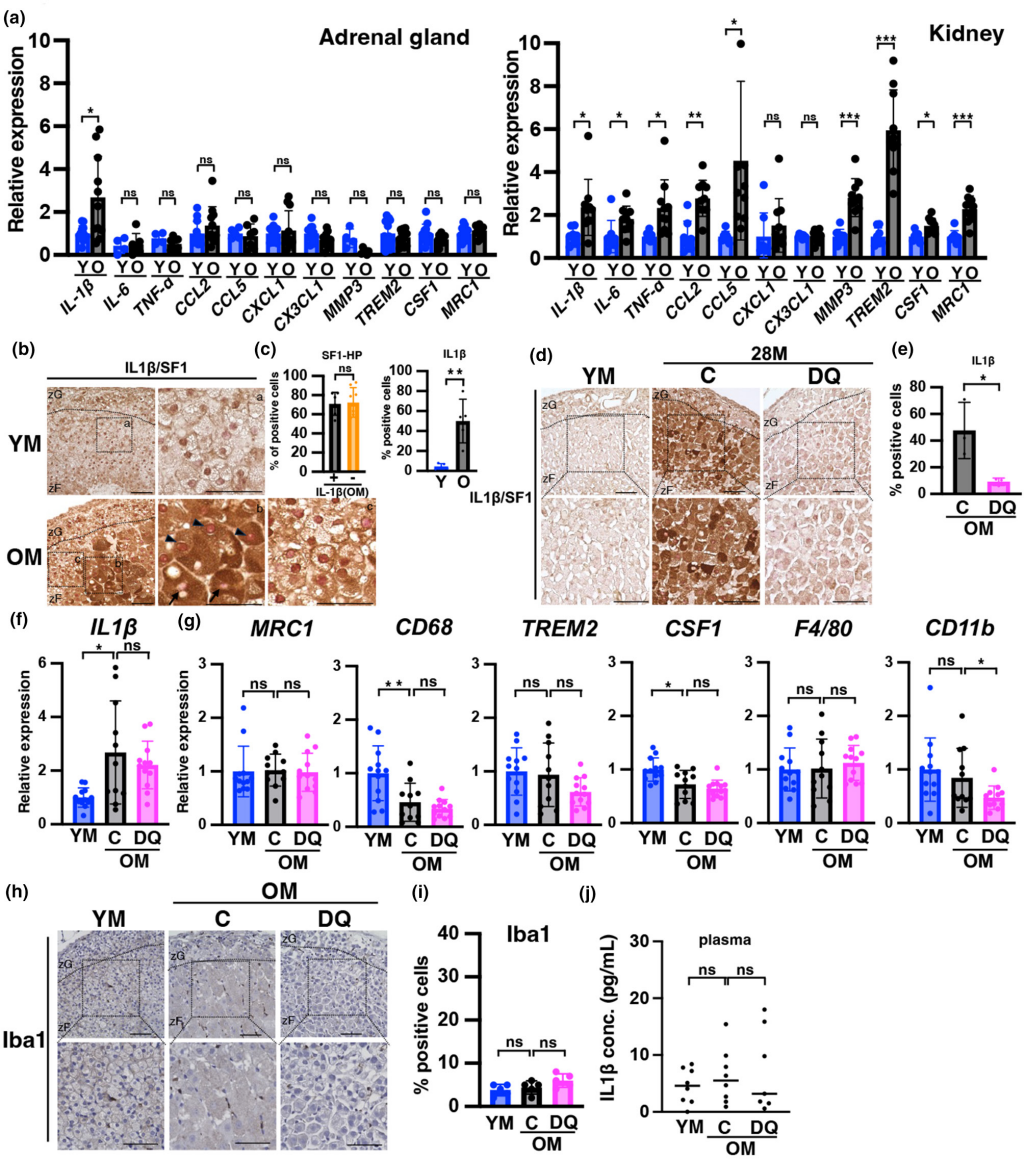
Expression of IL1β is induced in the aged adrenal zF, but without an increase in other SASPs or macrophage infiltration. (a) The mRNA expression levels of SASP factors in the adrenal cortex and kidney of YM and OM are shown. Data are presented as relative values and were normalized to TBP. (b, d, and h) Immunostaining of mice adrenal glands with IL1β (brown; b, d), SF1 (red; b, d), Iba1 (brown; h) in YM, OM, and DQ groups. h; nuclear stained (blue). Each section was stained with an anti‐IL1β, ‐SF1, and Iba1 antibodies and observed using a light microscope. Scale bar (solid line) = 50 μm. zF, zona fasciculata; zG, zona glomerulosa. (C: control, DQ: senolysis). (b) a, b, and c are enlarged views. Arrowheads indicate cells highly positive for SF1 (SF1‐HP); arrows indicate SF1‐negative cells. (c) Percentage of SF1‐HP cells among IL1β‐positive and ‐negative cells in zF (Left panel). (c, e, and i) Percentage of positive cells for IL1β (C; right panel, e) and Iba1 (i) in zF. (f and g) mRNA expression levels of *IL1β* and macrophage factors in the adrenal cortex in YM, OM, and DQ groups. IL1β data are presented as relative values and normalized to both TBP and β2‐microglobulin. (j) Measurements of IL1β levels in mouse plasma in YM, OM, and DQ groups. Data are shown as the mean ± SD. Asterisks indicate statistical significance (*p < 0.05, **p < 0.001, ***p < 0.0001, ns; not significant). The quantitative results of immunostaining are summarized in Table S1.

The results thus far indicate that accumulation of senescent cells is involved in the oversecretion of CORT. Therefore, we investigated if SASP is involved in CORT overproduction in adrenocortical senescent cells. mRNA levels of many SASP factors increased in the kidneys of OM, whereas only IL1β was upregulated in the adrenal cortex (Figure 4a). Therefore, we focused on IL1β and found that approximately half of the cells expressing low (as shown by arrows in Figure 4b) and high (as shown by arrowheads in Figure 4b) levels of SF1 in the adrenal zF, that is, adrenocortical cells, exhibited strong immunostaining for IL1β in OM, whereas few cells displayed this staining in YM (Figure 4b,c). In both IL1β‐positive and ‐negative cells, approximately 60% of the cells were SF1‐HP cells (Figure 4c). In DQ‐treated mice, we observed a few IL1β‐positive cells as well as SF1‐HP cells (Figure 4d,e), and mRNA levels tended to be suppressed by DQ treatment (Figure 4f). Next, we investigated macrophage infiltration. mRNA levels of macrophage markers, namely, *MRC1*, *CD68*, *TREM2*, *CSF1*, *F4/80*, and *CD11b*, did not increase in the OM or DQ group compared with those in YM (Figure 4g). Immunostaining of Iba1 in adrenal zFs showed no increase in the OM and DQ groups (Figure 4h,i). The level of IL1β in mouse plasma from YM, OM, and DQ groups was not significantly different (Figure 4j). The data suggest that IL1β secreted from senescent adrenocortical cells in the zF may affect neighboring cells in an autocrine and paracrine manner, potentially promoting excessive CORT secretion in aged mice.

### 2.5 Neutralization antibodies for IL1β inhibit accumulation of senescent cells in aged adrenal gland and CORT overproduction

**FIGURE 5:**
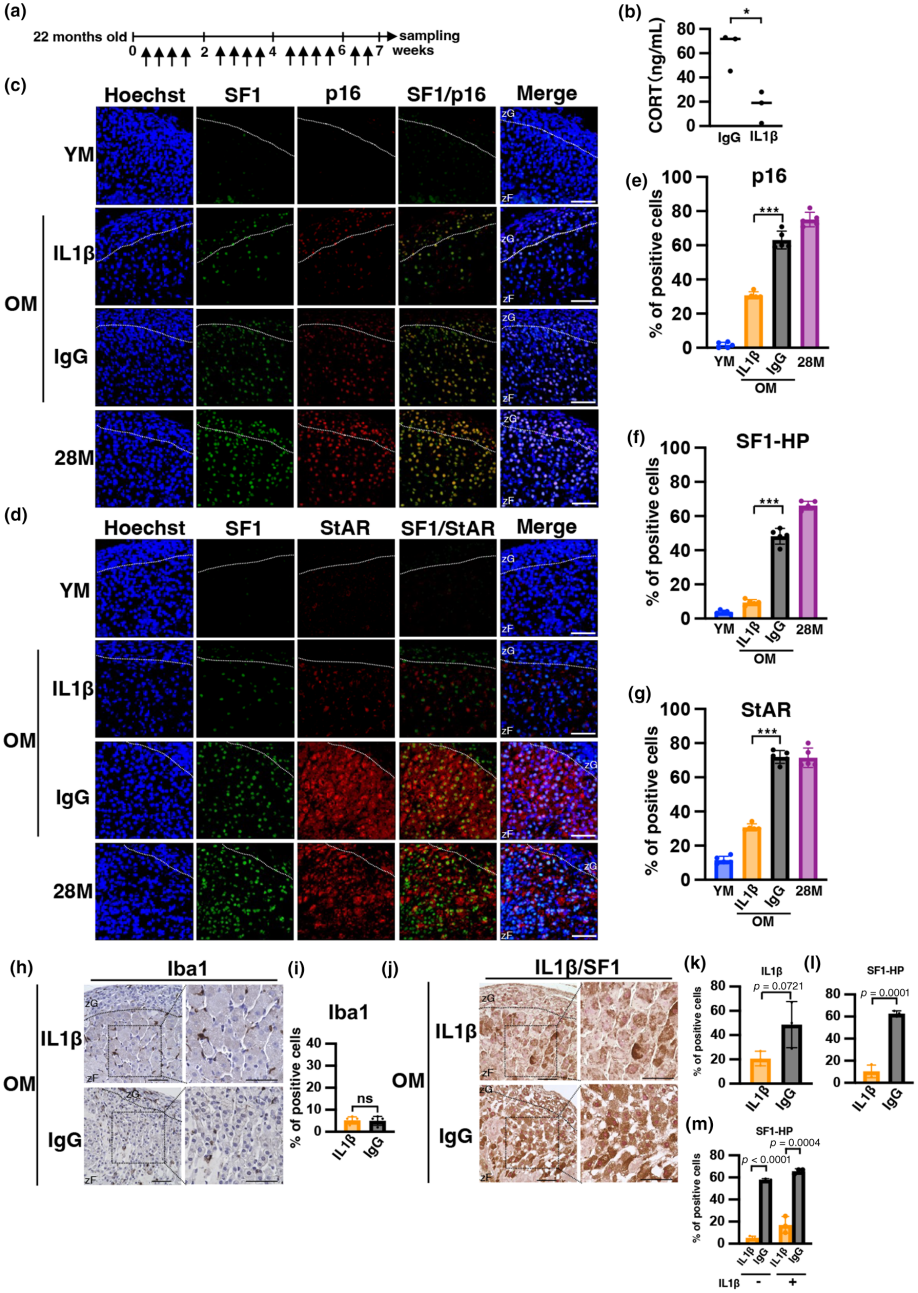
Neutralizing antibodies against IL1β inhibit hypersecretion of CORT along with adrenal aging in mice. (a) Schedule of IL1β‐antibody administration experiments. Arrows indicate antibody administration. (b) CORT levels at ZT0 treated with IgG and IL1β‐antibodies were determined. CORT levels were measured in plasma samples using ELISA. (c, d, h, and j) Immunofluorescence images of p16, SF1, StAR, Iba1, and IL1β in the adrenal gland of IL1β‐antibody‐treated mice. Each section was stained with anti‐p16, ‐SF1, ‐StAR, ‐Iba1, and IL1β antibodies and observed using confocal laser scanning microscopy and light microscopy. h indicates Iba1 in brown and nuclear in blue. j indicates IL1β in brown and SF1 in red. Scale bar (solid line) = 50 μm. zF, zona fasciculata; zG, zona glomerulosa. (YM: young mice, IL1β and IgG: antibody administered mice, OM: 24‐month‐old mice, 28 M: 28‐month‐old mice). (e–g, i, k, and l) Percentage of cells positive for p16, SF1‐HP, StAR, Iba1, and IL1β in zF. (m) The percentage of SF1‐HP among IL1β‐positive and IL1β‐negative cells in zF was determined in IgG and IL1β‐antibody. SF1‐HP; SF1‐high positive cells. Data are shown as the mean ± SD. Asterisks indicate statistical significance (*p < 0.05, ***p < 0.0001, ns; not significant). The quantitative results of immunostaining are summarized in Table S1.

To clarify the involvement of IL1β in excess CORT secretion in the OM group, mice were treated with IL1β antibodies to suppress the effects of IL1β. The experimental schedule is shown in Figure 5a. Plasma IL1β levels were completely suppressed in mice treated with IL1β antibodies (Figure S6a). Measurement of mouse plasma CORT levels at ZT0 revealed significant suppression in the IL1β‐treated group compared with the control group (Figure 5b). Immunostaining of the adrenal glands showed a decrease in p16‐, SF1‐HP, and StAR‐positive cells (Figure 5c–g) but no change in γH2AX‐positive cells (Figure S6b,c). Iba1 staining revealed no change in macrophage infiltration in the IL1β‐treated group (Figure 5h,i).

As shown in Figure 5j, the IL1β antibody‐treated group exhibited a tendency toward decreased expression of IL1β‐positive cells compared with the control IgG group (IgG 49% vs. IL1β 21%, *p* = 0.072), although this difference was not significant (Figure 5k). In contrast, a significant decrease was observed in the number of SF1‐HP cells (IgG 63% vs. IL1β 10%, *p* = 0.0001; Figure 5l). Particularly in the IL1β‐negative cells, the reduction of the SF1‐HP cells was highly significant (*p* < 0.0001; Figure 5m). These findings suggest that IL1β secreted from certain adrenocortical cells, potentially senescent cells, may influence neighboring cells and activate steroidogenesis as part of the SASP. Overall, these results indicate that IL1β is not only involved in the regulation of aging in the mouse adrenal gland but also in the expression levels of SF1, that is, CORT production.

### 2.6 Dexamethasone‐induced aging in the mouse adrenal gland

**FIGURE 6:**
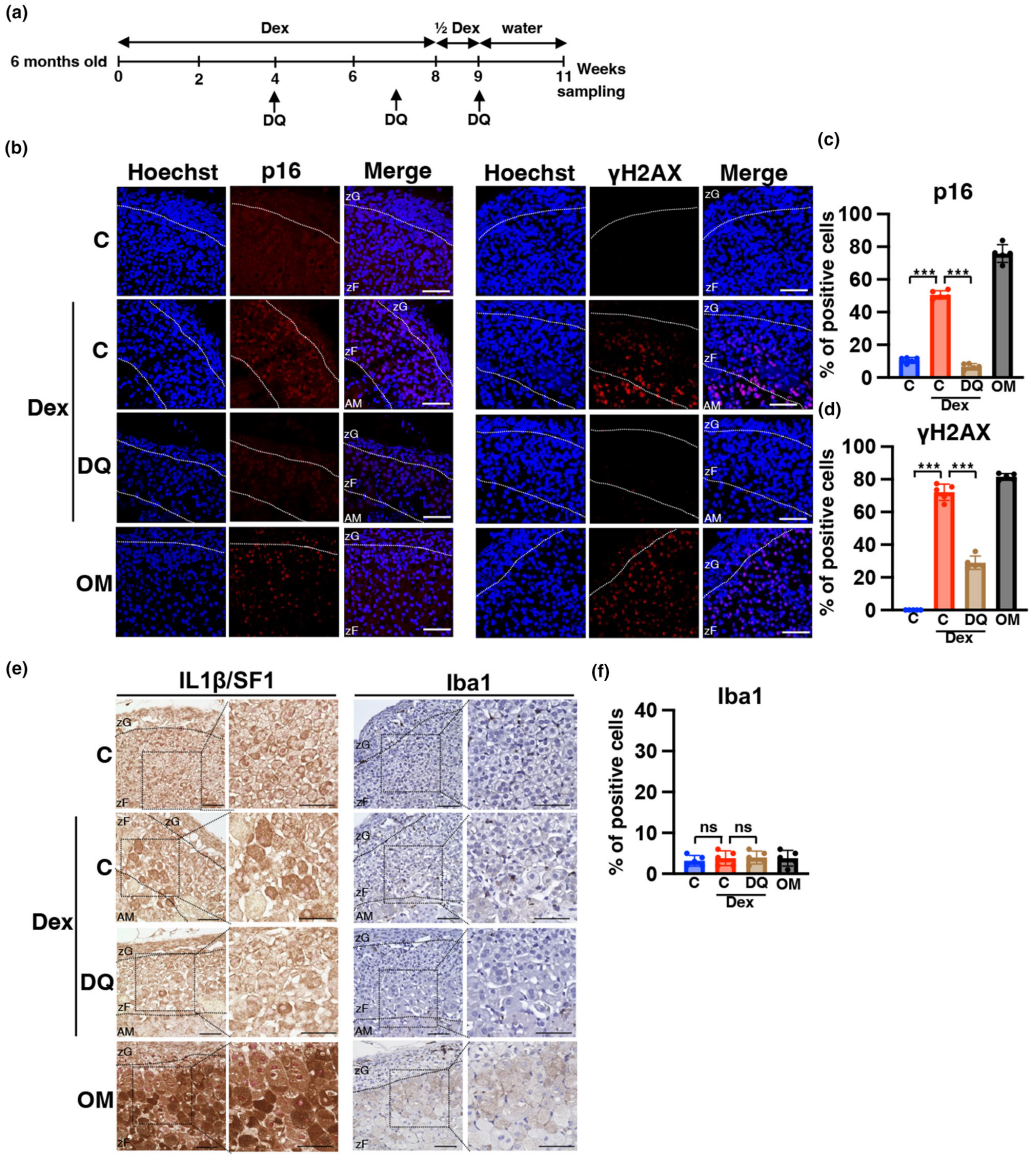
Chronic administration of dexamethason promotes accumulation of cells expressing senescense markers in the adrenal zF of younger mice. (a) Schedule of dexamethasone (Dex) administration experiments. (b and e) Immunofluorescence images of p16, γH2AX, IL1β, SF1, and Iba1 in the adrenal gland of Dex‐treated mice. Each section was stained with anti‐p16, ‐γH2AX, ‐IL1β, ‐SF1, and ‐Iba1 antibodies and observed using confocal laser scanning microscopy and light microscopy. Left part of e indicates IL1β in brown and SF1 in red. Right part of e indicates Iba1 in brown and nuclear in blue. Scale bar (solid line) = 50 μm. AM, adrenal medulla; zF, zona fasciculata; zG, zona glomerulosa (C, control mice; Dex, Dex‐treated mice; DQ, DQ‐treated mice; OM, old mice; YM, young mice). (c, d, and f) Percentage of cells positive for p16, γH2AX, and Iba1. Data are shown as the mean ± SD. Asterisks indicate statistical significance (***p < 0.0001, ns; not significant). The quantitative results of immunostaining are summarized in Table S1.

We have demonstrated that the diurnal variation in CORT concentration diminishes with aging, with levels remaining elevated throughout the day (Figure 1a,b). To investigate whether chronic administration of dexamethasone (Dex), a steroidal agent similar to corticosterone, can induce aging, we administered Dex via drinking water to 6‐month‐old mice, following the schedule outlined in Figure 6a. We observed atrophy of adrenal zFs, with the rate of p16‐ or γH2AX‐positive cells in atrophic zFs increasing to an extent similar to that observed in aged mice (Figure 6b–d). Immunostaining of IL1β and Iba1 in the adrenal glands of mice administered Dex showed some cells expressing IL1β, albeit less strongly than their expression in OM, accompanied by no increase in Iba1‐positive cells (Figure 6e,f). In summary, chronic administration of GC induced at least some phenotypes of aging.

### 2.7 CORT‐producing system differs in aged female and male mice

In YM, females had diurnal rhythms at higher CORT concentrations than males (Figure S1). In OM, CORT at ZT0 was significantly lower in females than in males, maintaining the diurnal rhythm (Figure 1a, Figure S1; male vs. female, *p* = 0.0009, Figure S7a). Thus, adrenal cortexes obtained at ZT0 were used to analyze differences in zFs between males and females. Notably, SF1‐HP cells were rarely observed in OM (Figure S7b). A certain number of p16‐, γH2AX, or IL1β‐positive cells were observed in OM (Figure S7b,c, Figure S8a–c), although lower than the number observed in males, such cells were significantly reduced in the DQ group (Figure S9a–c). This indicated the accumulation of senescent cells in the adrenal zF with aging in females, albeit to a lesser extent than in males. The intensity and number of IL1β‐positive cells were also lower in females compared to males of the same age and almost disappeared after DQ administration (Figure S7c and S9d,e). Additionally, the percentage of Iba1‐stained cells significantly increased in females, reaching approximately 10% in OM, and decreased in the DQ group to a level comparable to YM, whereas no such increase was observed in males (Figure S7d). In summary, females exhibited less pronounced IL1β expression and senescent cell accumulation than males. They also differed from males in the absence of strong SF1 expression, CORT hypersecretion, and in the presence of mild increase in macrophage infiltrate.

## 3 DISCUSSION

GC hypersecretion increases susceptibility to aging‐related diseases (Gassen et al., 2017). In this study, we found that while in YM, CORT blood levels at ZT0 and ZT12 represented the nadir and the peak of circadian GC oscillations, respectively, they remained high throughout the day in OM, resulting in the disappearance of the diurnal variation in male (Figure 1a and Figure S1). Concomitantly, the adrenal glands accumulated senescent cells, and the administration of senolytics, dasatinib (D), and quercetin (Q), suppressed increased‐CORT production at ZT0 associated with aging, accompanied with decreased senescent cells in adrenal zFs. We further found that one of SASP factors, IL1β highly expressed in adrenocortical cells in OM, and the inhibition of IL1β by administration of neutralizing antibody against IL1β also decreased CORT production at ZT0 in OM. Collectively, these suggest that the increase in GC secretion in OM is due to the secretion of IL1β from accumulated senescent adrenal cells. This is the first report to demonstrate that senescent cells promote GC hypersecretion.

### 3.1 Increased GC secretion and decreased diurnal variation in OM

We examined blood levels of CORT in the morning (ZT0) at different ages (in months) and found that the nadir level of CORT started to increase at the age of 18 M and further increased to the same as the peak time point of the day (ZT12) at 24 M in male mice (Figure 1b). Yau et al. reported that nadir CORT levels significantly increased in 18 M–20 M mice compared with 4 M–7 M mice (Yau et al., 2001). Because the increase in morning (ZT0) CORT in OM compared with YM was larger than in evening (ZT12), the diurnal range of variation disappeared. In humans, the level of cortisol, a main GC for human, in the nocturnal nadir increased progressively with aging. Although the diurnal rhythmicity of cortisol secretion was preserved in old age, the relative amplitude was dampened (Ferrari et al., 2001); (Purnell et al., 2004); (Van Cauter et al., 1996) (Van Cauter et al., 2000). These findings suggest that the persistence of high CORT levels throughout the day and the decrease in diurnal variation are common features of aging in humans and mice. Based on our data, we hypothesize that the elevation of the nadir CORT levels may be a characteristic of the late‐stage elderly.

### 3.2 Age‐related changes in adrenal glands

Previous reports have shown that excess GC secretion from human cortisol‐secreting tumors promotes cellular senescence in adrenal glands (Pieroni et al., 2021). Increased expression of p21 has been observed in tissue damaged by 30 min of ischemia in the adrenal glands of rats and humans following surgery (Didenko et al., 1996). Telomere shortening is generally considered an aging‐related phenomenon, and a similar phenotype was observed with aging in human male adrenal glands (Nonaka et al., 2020). Our study showing the accumulation of senescent cells in the adrenal glands is the first to observe adrenal aging at the molecular and cellular levels in naturally aging mice. We observed an accumulation of cells in the adrenal glands of OM with the previously known markers of senescent cells, including p16, γH2AX, 53BP1, and β‐galactosidase expression as well as cell enlargement (Aguayo‐Mazzucato et al., 2017); (De Cecco et al., 2011) (He & Sharpless, 2017) (Kreiling et al., 2011). Analysis of GC‐producing tissues (zF) by age in months indicated that senescent cells are observed from 18 M and approximately 80% of cells in the zF at 24 M exhibited a senescent phenotype, indicating that senescent cells accumulate with age. Morning GC secretion begins to rise at the same monthly age as senescent cells begin to appear in the zF. GC is further elevated with an increase in the number of senescent cells, suggesting that senescence in the zF may be involved in morning nadir GC secretion.

### 3.3 Improvement of GC production system by senolytics

Senolysis induces selective cell death in senescent cells by exploiting the differences in the biological characteristics between senescent and normal cells. There are multiple mechanisms of senescence progression in vivo and treatment focuses on the phenomena that promote tissue aging. Dasatinib (D) and quercetin (Q), which represent the first senolytic drugs that induce cell death in senescent cells, were discovered because senescent cells are predisposed to apoptosis (Zhu et al., 2015). The administration of dasatinib (D) and quercetin (Q) to OM delays the onset of osteoporosis and other geriatric diseases and extends longevity (Farr et al., 2017). The elimination of senescent cells in genetically modified mice has many beneficial effects and the development of senolytic drugs to induce senescent cells to selective cell death is an active area of research. The number of senescent cells that express p16, γH2AX, and 53BP1 as well as the area of β‐galactosidase staining was significantly reduced in the adrenal zF of D‐ and Q‐treated OM, indicating that senescent cells were removed. Concomitantly, CORT blood levels at ZT0 were markedly decreased to levels similar to that of YM, and the diurnal amplitude of CORT was restored in the treated OM (Figure 3b). Similarly, the expression of *SF1* and *CYP11B1*, an enzyme that converts cholesterol to CORT specifically expressed in the adrenal zF, were reduced to YM levels (Figure 3e). Immunohistological analysis also indicated markedly reduction of p16‐ and SF1‐HP cells (Figure 3f–h). These data provide further evidence that senescent cells are involved in the overproduction of CORT at ZT0 in OM. Notably, the thickness of the adrenal zF does not thin and the number of cells does not appear to decrease, even though senolytics remove senescent cells, which constitute the majority in the OM adrenal zF. In the DQ group, *caspase9* mRNA levels were unchanged, TUNEL signal did not increase, and Ki67 was unchanged, suggesting that neither cell death nor proliferation was notably induced in adrenal zFs (Figure S10a–c). Experiments with neutralizing antibodies for IL1β suggest that IL1β promotes cellular senescence. In addition, Dex induced cellular senescence, suggesting that GC is an SASP in adrenal zFs. Thus, we hypothesized that the initial senescent cells induce surrounding cells to enter a state of senescence via the secretion of IL1β and CORT. It is presumed that DQ administration was initiated when senescent cells were scarce, thereby preventing the subsequent emergence of such cells. As a result, despite the removal of senescent cells, there was no notable decrease in the cell numbers of the adrenal zF.

### 3.4 Mechanisms of enhanced GC production in the old adrenal gland

In this study, we found that the expression of IL1β, one of the main SASPs, increased in adrenal zF cells in OM. In addition, inhibition of IL1β by administration of a neutralizing antibody against IL1β ameliorated CORT hypersecretion. Histological analysis revealed a reduction in senescent cells in the adrenal zFs following the treatment. IL1β‐induced cellular senescence in other cellular systems (Shang et al., 2020), and our data suggest that in adrenal tissue, aging adrenal cells secrete IL1β and promote senescence in surrounding cells. Furthermore, treatment with an IL1β‐neutralizing antibody, despite the retention of some IL1β‐positive or senescent cells, led to a marked reduction in SF1‐HP cells, suggesting that secreted IL1β may contribute to the high expression of SF1 in the adrenocortical cells of OM. This finding is supported by previous studies demonstrating that IL1β promotes GC secretion in cultured human H295R adrenocortical cells and rats (Tkachenko et al., 2011); (Zieleniewski et al., 1995). Thus, we believe that IL1β acts directly on adrenocortical cells and promotes strong expression of SF1, and the subsequent secretion of CORT in OM. Possible origins of IL1β include circulating plasma and infiltrating inflammatory cells, in addition to local senescent adrenocortical cells in zFs. However, blood levels of IL1β were not significantly elevated in aged individuals (Figure 4j). A mild inflammatory cell infiltrate was observed in adrenal tissue, but not significantly increased in GC‐secreting zFs (Figure 4h,i). Thus, it is possible that IL1β secreted by senescent adrenocortical cells promotes GC secretion by affecting surrounding adrenocortical cells in OM in an autocrine or paracrine manner. In summary, senescent adrenal cells secrete IL1β, which affects surrounding cells, promotes CORT secretion, and induces senescence.

### 3.5 Another mechanism of morning (ZT0) GC hypersecretion

We examined other processes in addition to SF1 as potential mechanisms of morning GC hypersecretion during aging. The steroid‐related factor StAR transfers cholesterol from the mitochondrial inner membrane to the outer membrane (Son et al., 2008). The mRNA expression of *StAR* in YM exhibited clear diurnal oscillation, whereas in OM, the ZT0 nadir levels increased and the ZT12 peak levels decreased, resulting in the complete disappearance of diurnal variation (Figure 1e). From the experiment showing the removal of senescent cells by DQ administration, the nadir level of *StAR* and *CYP11A1* expression was decreased by D + Q treatment, but a significant difference was not obtained (Figure 3e). *StAR* and *CYP11A1* are known to be regulated not only by *SF1*, but also by the clock gene *Bmal* (Chen et al., 2013) (Son et al., 2008). Therefore, we measured the expression of clock genes using the same RNA samples as in Figure 1e. *Clock* and *Bmal*, as transcription factors of the bHLH‐PAS, play a central role in circadian clock oscillations. *Clock* and *Bmal* form a heterodimer and bind to a DNA cis sequence known as the E‐box. The E‐box‐dependent transcription mechanism of clock genes regulates the transcriptional activity of Period 2 (*per2*) and Cryptochrome 1 (*CRY1*). The expression of *Bmal*, *per2*, *Clock*, and *CRY1* was measured and diurnal variation, which was calculated the differences between in ZT0 and ZT12, was reduced in OM compared with YM for all genes, suggesting that clock gene expression may be affected by aging (Figure S11a). In most tissues, including the adrenal glands, the number of genes with diurnal variation in expression has been reported to decrease with age, indicating weakened circadian control (Wolff et al., 2023). Thus, we expected that rejuvenation through clearance of senescent cells would improve clock gene oscillation. However, contrary to expectations, when senescent cells were removed, the mRNA level of the clock genes was not repressed by DQ (Figure S11b). These results suggest that DQ does not respond to the suprachiasmatic nucleus (SCN) or does not reach the SCN through the blood–brain barrier. Therefore, the clock genes are not affected, which indicates that StAR and CYP11A1 expression may not be sufficiently reduced. Thus, SF1‐independent, clock gene‐dependent expression of StAR and CYP11A1 may be involved, albeit partially, in morning CORT hypersecretion in OM. Taken together, our results suggest that the main factor in aging‐related CORT hypersecretion is increased expression of SF1, whereas SF1‐independent StAR and CYP11A1 expression may also be partially involved.

### 3.6 Dex promotes cellular senescence in the aged adrenal cortex

In our study, chronic administration of Dex to YM resulted in the accumulation of cells with increased expression of the senescence phenotypes p16, and γH2AX (Figure 6b–f). GCs induced cellular senescence in other cell types (Poulsen et al., 2014) (Wang et al., 2021) (Tong & Chen, 2023). Consistently, our data indicate that GCs promote senescence in adrenocortical zFs. Therefore, we speculate that when even one senescent cell appears in the adrenal gland and GC secretion increases, cellular senescence is induced in the neighboring cells, increasing the number of senescent cells, that is, GC can be regarded as an SASP in the adrenal cortex. These findings suggest that increased GC secretion in the aging adrenal gland can further accelerate adrenal aging. However, questions regarding why the expression of an inflammatory SASP factor (IL1β) and macrophage infiltration are rarely induced remain. This could be attributed to the expression of most inflammatory SASPs involving NF‐kB activity (Liu et al., 2017), which is inhibited by the GC–GR complex, leading to the suppression of SASPs and inflammation. The lack of increase in inflammatory SASPs other than IL1β in the adrenal cortex of aged mice is speculated to be due to the excess secretion of GC. It is likely that GC overproduction locally maintains low levels of SASP in the aged adrenal gland.

### 3.7 Diurnal rhythm of CORT was maintained in female OM

In this study, we show that the GC diurnal rhythm in males disappears with aging, but is maintained in females. In female YM, SF1 is strongly expressed at ZT0, whereas few cells in aged mice strongly express SF1. Takahashi et al. showed that DHT suppresses SF1 expression and CORT is lower in males than females, and reported differences in the regulation of CORT production, as well as regulation of SF1 expression, in males and females (Takahashi et al., 2024). The low CORT at ZT0 in older female mice may be due in part to the expression of SF1. Compared to male mice, there were fewer cellular senescence markers, p16‐, and γH2AX‐, as well as IL1β‐, positive cells, whereas the degree of inflammatory cell infiltration was mildly but significantly increased (Figure S7b–d). Recently, Warde et al., Wilmouth et al., and Lyraki et al. reported that adrenal hyperplasia or tumorigenesis occur in Wnt‐activated mouse lines; however, males had a better prognosis, because cellular senescence in tumors is induced earlier in males than females, and tumor cells are eliminated by infiltrating inflammatory cells (Warde, Smith, Liu, et al., 2023) (Wilmouth Jr. et al., 2022) (Lyraki et al., 2023) (Warde, Smith, & Basham, 2023). Our natural aging model aligns with these findings, indicating the accumulation of fewer senescent cells in the zF of females compared with males. Consequently, induction of IL1β expression is weaker in females, preventing CORT hypersecretion. Furthermore, the induction of inflammatory cell infiltration is clearly observed in females (Figure S8d). This is likely due to the apparent lack of GC oversecretion. In summary, the limited accumulation of senescent cells may lead to inadequate induction of IL1β expression, potentially inhibiting CORT production and facilitating macrophage infiltration. However, the present study fails to fully explain the evident sex difference observed. This remains a subject for future investigation.

## 4 CONCLUSION

The findings reveal that same amount of CORT is secreted as that of the peak secretion time in old mice during the time of day when CORT is minimally secreted in young mice, resulting in disappearance of circadian rhythm. This is caused by a SASP, IL1β, secreted from senescent adrenocortical cells. In this study, no experiments on primates or humans were conducted. Although these are limitations, the study is significant because it clarified the underlying mechanism of adrenal senescence. Future studies will analyze the effects on aging and the development of aging‐related diseases.

## 5 METHODS

### 5.1 Animals

This study was approved by the Teikyo University Central Laboratory Animal Facility (approval 20‐028). All experiments were conducted in accordance with approved guidelines. Mice (C57BL/6J) and were purchased from Charles River Laboratories and NCGG Aging Farm (Aichi, Japan). The mice were maintained at 22°C ± 2°C on a 12:12‐h reverse light/dark cycle, with the dark period starting at 8 AM. Mice were housed in ventilated cages and maintained within a pathogen‐free facility with free access to a certified diet (standard mouse diet, CRF‐1, Charles River) and water. C57BL/6J breeding colonies were maintained to generate animals. We randomly assigned 20‐month‐old C57BL/6J mice to once‐monthly treatments by oral gavage of dasatinib and quercetin (D + Q) or vehicle for 4 months. D and Q were diluted in 10% PEG400 and administered at doses of 5 and 50 mg/kg, respectively, in 100 μL of solution. Neutralizing antibodies for IL1β were in vivo Mab anti‐mouse/rat IL1β (200 μg per mouse, BioXCell, BE0246) and in vivo Mab polyclonal Armenian hamster IgG (200 μg per mouse, BioXCell, BE0091) for controls, administered intraperitoneally twice weekly for 7 weeks. Dexamethasone loading tests were conducted using water‐soluble dexamethasone (Sigma, D2915), adjusted to 0.0028 mg/mL in water, provided ad libitum for 8 weeks, followed by 0.0014 mg/mL dexamethasone for 1 week. The mice were then returned to water for 2 weeks.

### 5.2 Tissue collection

Mice were sacrificed in the morning (ZT0), daylight (ZT6), evening (ZT12), and late‐evening (ZT18). Body mass was recorded and blood was collected via the tail and abdominal veins at the time of death and stored at −80°C. Blood sampling from the tail vein was performed within 15–30 s to reduce stress on the mice. The adrenal glands and liver were embedded in optimal cutting temperature compounds and stored at −80°C.

### 5.3 RNA extraction from adrenal cortex by laser microdissection

Cryosections (30 μm) were prepared and dehydrated in graded ethanol solutions (70% once, 1 min, 80% once, 1 min, 90% twice, 1 min, 100% twice, 1 min). After air‐drying for 5 min, laser capture was performed under direct microscopic visualization of the adrenal cortex (only the zona fasciculata region, excluding the zona glomerulosa of the adrenal cortex) areas by melting the selected regions onto a thermoplastic film mounted on optically transparent laser capture microdissection caps (LMD6, Leica). The LMD6 System was set to the following parameters: power: 60, aperture: 20, speed: 15, and specimen balance: 10. The transfer film was examined under a microscope to ensure complete cell lysis. Total RNA was extracted using a phenol–chloroform‐based method (RNA iso Plus, Takara Bio, Inc.) and using the RNeasy Micro Kit (Qiagen) based on the manufacturer's instructions with some minor modifications. To eliminate potential genomic DNA contamination, RNA samples were treated with DNase I (RNase‐Free DNase Set, QIAGEN) at 25°C for 15 min.

### 5.4 Analysis of gene expression by quantitative RT‐PCR (RT‐qPCR)

First‐strand cDNA was prepared using random hexamers and synthesized from 100 ng of total RNA. The PrimeScript™ RT reagent kit (Takara Bio, Inc.) was used for reverse transcription. Quantitative PCR was performed using the Thermal Cycler Dice® Real Time System III (Takara Bio Inc.) and the recommended cycling parameters, which included 1 μL cDNA diluted 3‐fold with EASY Dilution solution (Takara Bio Inc.) and TB Green® Premix Ex Taq TMII (Takara Bio, Inc.). The data were normalized to the expression of TATA‐binding protein and β2 microglobulin. The primer sequences for each gene are listed in Table S2.

### 5.5 Analysis of corticosterone, ACTH, and IL1β using ELISA

Corticosterone (ADI‐900‐097, Enzo Life Sciences), ACTH (ab263880, abcam), and IL1β (KE10003, Proteintech Group, Inc.) levels were measured by ELISA based on the manufacturer's instructions. The optical density (OD) was read on an ELISA Multiscan Plus plate reader at 405 or 450 nm. Negative control values resulting from the addition of mouse serum were subtracted from the sample OD values. Each measurement was performed in duplicate.

### 5.6 Fluorescence immunostaining

Cryosections (10 μm) were fixed with 4% paraformaldehyde in phosphate‐buffered saline (PBS) for 20 min. The fixed sections were permeabilized with 0.2% Triton X‐100 in PBS for 20 min. After treatment with 0.1% Triton X‐100 in PBS and 5% bovine serum albumin (BSA), 0.2% goat serum, and 0.2% donkey serum for 1 h, the sections were incubated with primary antibody overnight at 4°C, as detailed in Table S3. After incubation, secondary antibodies conjugated with Alexa 647 or Cy‐3 was added for 2 h at 25°C as detailed in Table S3. Nuclei were stained using Hoechst 33258 (Invitrogen). The slides were mounted using Dako fluorescent mounting medium (Dako UK Ltd., Ely, UK) and examined using a fluorescence microscope (BZ‐X800, KEYENCE), ZEISS LSM 880 confocal laser scanning microscope (ZEISS), and Nikon A1si confocal laser scanning microscope (Nikon, Tokyo, Japan). Nikon NIS‐Elements and Image J were used to quantify the signal.

### 5.7 Senescence‐associated β‐galactosidase (SA‐β‐gal) assay

SA‐β‐gal staining was performed for the mouse adrenal glands and liver. Cryosections were fixed with 2% formaldehyde (Sigma‐Aldrich) in PBS for 5 min. After washing with PBS three times, the samples were incubated in SA‐β‐gal solution (pH 6.0) at 37°C for 16–18 h. Ice‐cold PBS was used to stop the reaction. In blinded analyses, for each sample, 10 images were acquired from random fields using fluorescence microscopy (BZ‐X800, KEYENCE).

### 5.8 Immunohistochemistry

Adrenal glands obtained from mice were fixed in 4% paraformaldehyde. Fixation was by immersion or perfusion. The fixed samples were prepared by Genostaff Co., Ltd. (Tokyo, Japan) as paraffin blocks and sections (6 μm). Sample sections were deparaffinized and antigen‐activated (70°C for 20 min) in citrate buffer (10 mM; pH 6.0). Endogenous enzymes were inactivated by BIOXALL (Vector) and reacted with primary antibodies. ImmPACT Vector Red AP Substrate kit and ImmPRESS HRP Horse Anti‐Rabbit IgG PLUS Polymer Kit were used for secondary antibodies and chromogenic substrates. In blinded analyses, for each sample, 10 images were acquired from random fields using bright‐field microscopy (BZ‐X800, KEYENCE).

### 5.9 TUNEL and H&E staining

Adrenal glands obtained from mice were fixed in 4% paraformaldehyde. Histopathological analysis of the adrenal glands was performed after H&E and TUNEL staining (Genostaff Co., Ltd., Tokyo, Japan). TUNEL staining was performed using in situ Apoptosis Detection Kit (Takara MK500). The area of the cells was measured using the ImageJ software.

### 5.10 Statistical analysis

Quantitative data are expressed as the mean ± standard error. Statistical analysis was performed using Student's *t*‐test or one‐way analysis of variance followed by a post hoc Turkey's test using the GraphPad Prism 10 software.

## AUTHOR CONTRIBUTIONS

N.O, T.S, M.A, H.H, J. A, Noboru. O, and M.T‐A performed experiments using mouse. N.O and Mi‐Ho A performed RT‐qPCR. N.O performed immunohistochemistry. N.O and M.T‐A were involved in the preparation of the manuscript. M.I, H.O, and T.O designed experiments. All authors read and approved the final manuscript.

## FUNDING INFORMATION

This research was supported in part by the JSPS KAKENHI Grant Numbers (No. 19K11651 and 22 K11788 to M.T‐A, No. 20K05943 and 23K10926 to N.O, and No. 17K09891 to T.O).

## CONFLICT OF INTEREST STATEMENT

All authors declare that they have no competing interest for the current work.

## Supporting information


Figures S1–S11

Tables S1–S3

## Data Availability

The data that support the findings of this study are available from the corresponding author upon reasonable request.
