# Supplementary material for: Accumulation of senescent cells in the adrenal gland induces hypersecretion of corticosterone via IL1β secretion
Source: Aging Cell. 2024 May 20;23(9):e14206. doi: 10.1111/acel.14206 (PMC11488315; doi:10.1111/acel.14206)
Supplement: Supplementary file 1 — Figures S1–S11 [file ACEL-23-e14206-s002.pdf]

Supplementary Figure 1

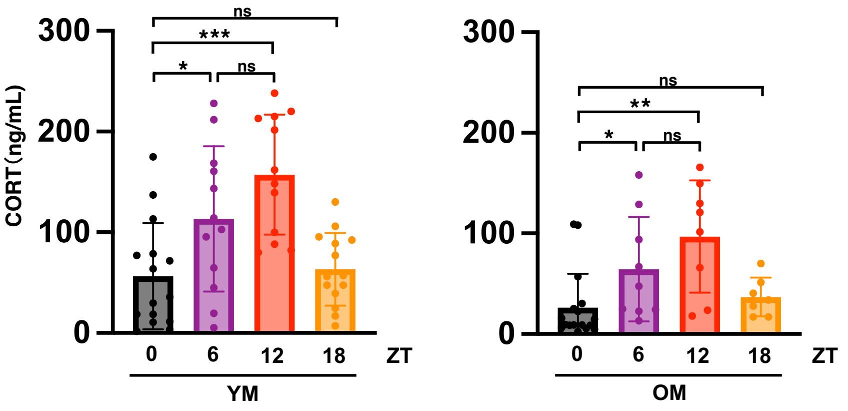

Figure S1. CORT levels in mouse plasma samples obtained every 6 h (ZT0, 6, 12, 18; 08:00, 14:00, 20:00, 2:00, lights on: ZT0, lights off: ZT12) in 6-month-old female mice (YM) and ≥20-month-old female mice (OM) in diurnal rhythm. Data are shown as the mean ± SD. Asterisks indicate statistical significance (**P* < 0.05, ***P* < 0.001, ****P* < 0.0001, ns; not significant).

Supplementary Figure 2

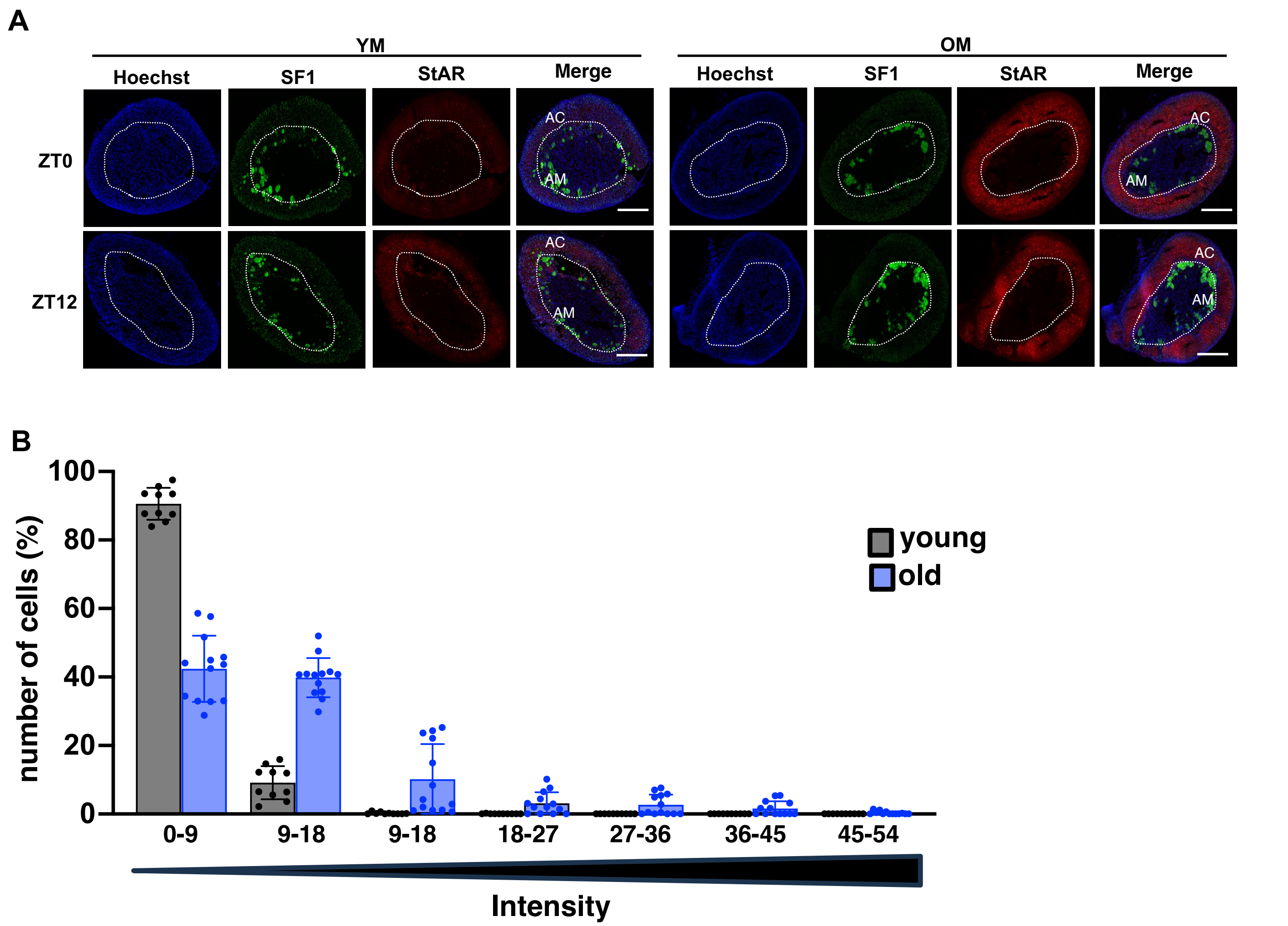

Figure S2. A. Immunofluorescence analysis of SF1 and StAR in the adrenal gland in YM and OM at ZT0 and ZT12, respectively, using fluorescence microscopy. Scale bar = 500 µm. AC: adrenal cortex, AM: adrenal medulla. **B**. Histogram showing fluorescence intensity criteria for SF1-positive cells. The criterion for SF1-high positive cells (SF1-HP) was determined as cells with intensity greater than "9." Fluorescence intensity of SF1 was corrected for Hoechst.

Supplementary Figure 3

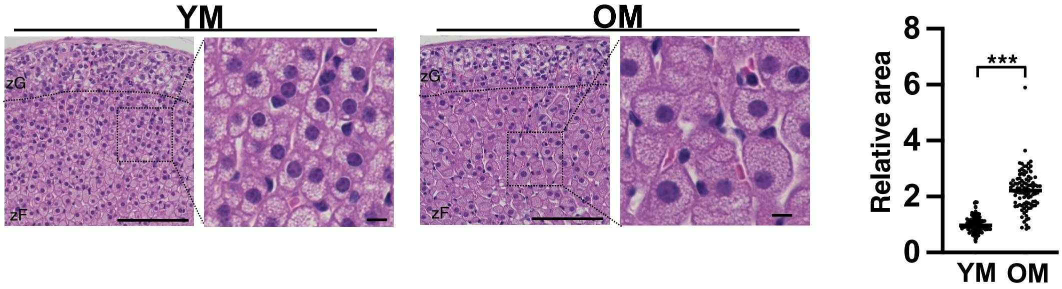

Figure S3. Tissue sections prepared from YM and OM (female) adrenal glands were stained with hematoxylin–eosin (H&E) for histological evaluation and assessed with light microscopy. Scale bar (solid line) = 50 µm. zG: zona glomerulosa, zF: zona fasciculata. The size of cells in H&E-stained adrenal glands was quantified in YM and OM.

Supplementary Figure 4

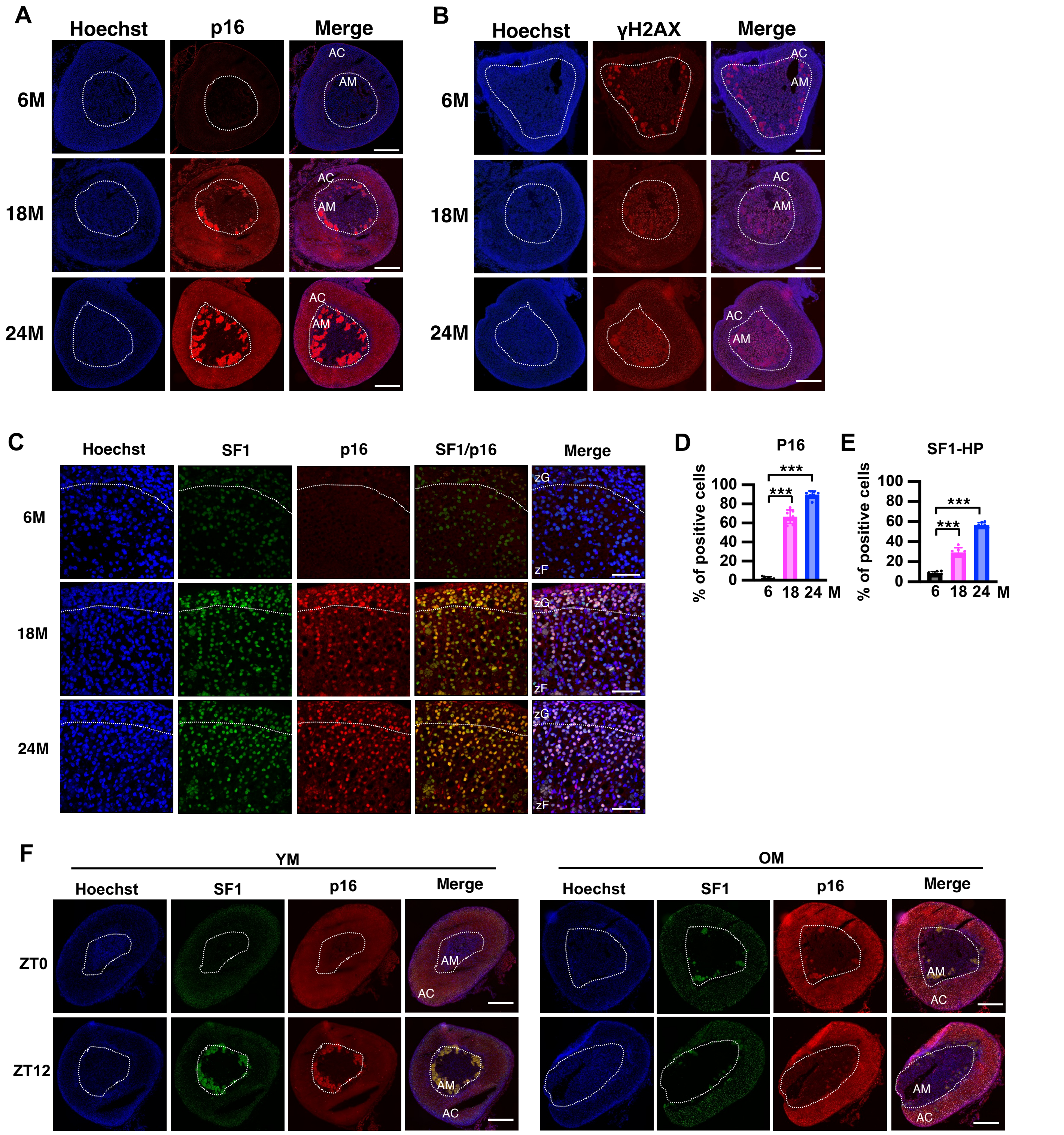

Figure S4. A and B. Immunofluorescent analysis of p16 and γH2AX in the whole adrenal gland at 6, 18, and 24 months. Each section was stained with an anti-p16 and γH2AX antibody and observed using a fluorescence microscopy. Scale bar = 500 μm. AC: adrenal cortex, AM: adrenal medulla. **C.** Immunofluorescent analysis of p16 and SF1 in the adrenal gland at 6, 18, and 24M. Each section was stained with an anti-p16 and SF1 antibody and observed using a confocal laser scanning microscope. Scale bar = 50 μm. zG: zona glomerulosa, zF: zona fasciculata. **D and E.** The percentage of p16- and SF1-positive cells (SF1-HP) in zF was quantified at 6, 18, and 24M. Cell counts were calculated from photographs, including those in Figure S4 D. **F.** Immunofluorescence analysis of p16 and SF1 using a fluorescence microscopy in the adrenal gland of YM and OM at ZT0 and ZT12. Scale bar = 500 μm. Data are shown as the mean ± SD. Asterisks indicate statistical significance (***$P < 0.0001$). The quantitative results of immunostaining are summarized in Table S1.

Supplementary Figure 5

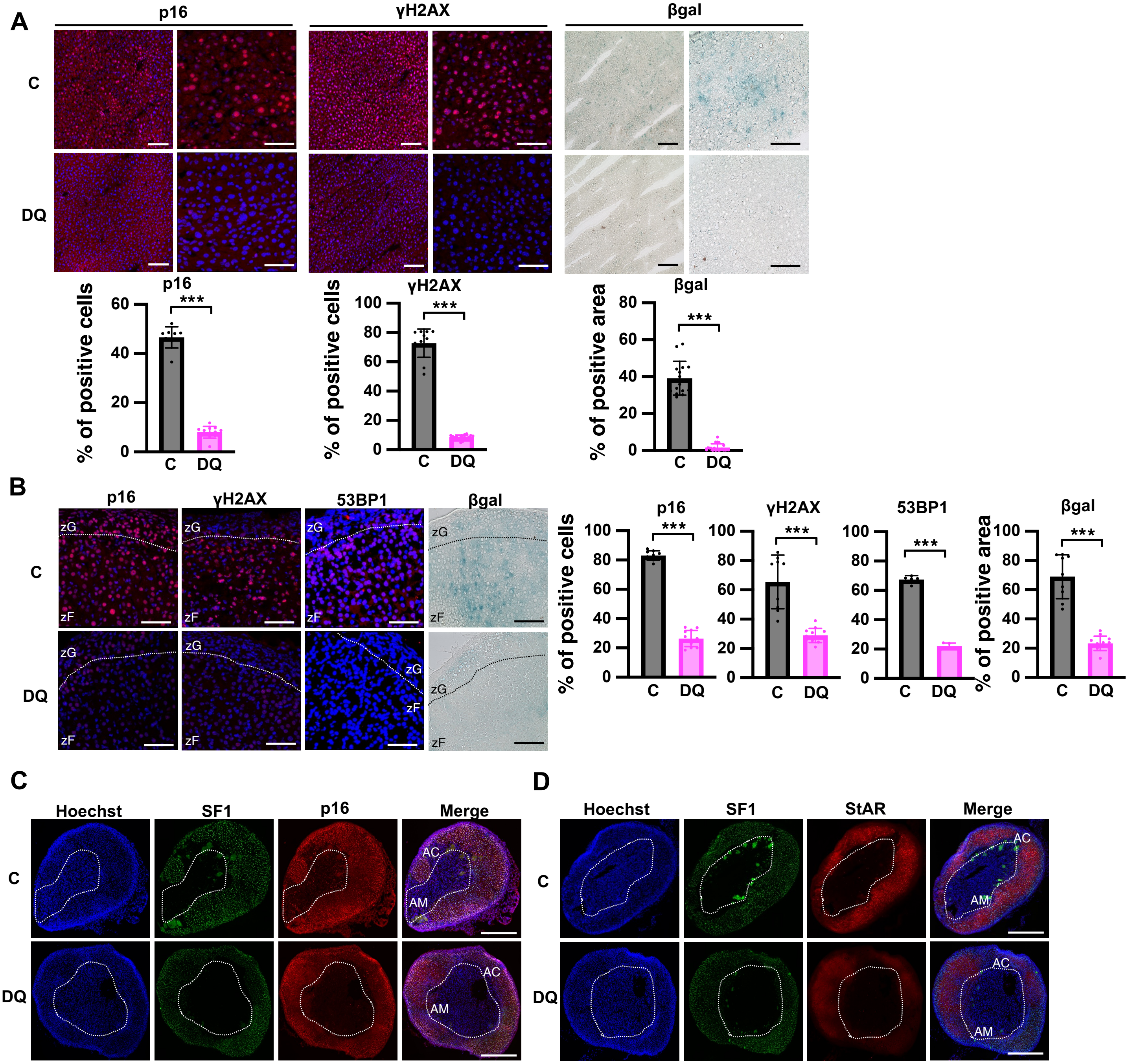

Figure S5. A. The expression of senescence markers in the liver was reduced by treatment with senolytic drugs. Each section was stained with anti-p16 or -γH2AX antibodies. β-galactosidase activity was tested using X-gal as substrate and observed using confocal laser scanning microscopy and light microscopy. Scale bar (solid line) = 50 μm. **B.** Immunofluorescence analysis of p16, γH2AX, and 53BP1, and as well as β-galactosidase activity, in the adrenal gland in dasatinib + quercetin (DQ)-treated mice. Figure 3F shows the results of immunostaining in different mice. Scale bar (solid line) = 50 μm, zG: zona glomerulosa, zF: zona fasciculata. **C and D.** Immunofluorescence analysis of p16, SF1, and StAR in the adrenal gland of DQ-treated mice. Each section was stained with anti-p16, -SF1, and -StAR antibodies and observed using a fluorescence microscopy. Scale bar = 500 μm. AC: adrenal cortex, AM: adrenal medulla. (C: control, DQ: senolysis). Data are shown as the mean ± SD. Asterisks indicate statistical significance (***P < 0.0001). The quantitative results of immunostaining are summarized in Table S1.

Supplementary Figure 6

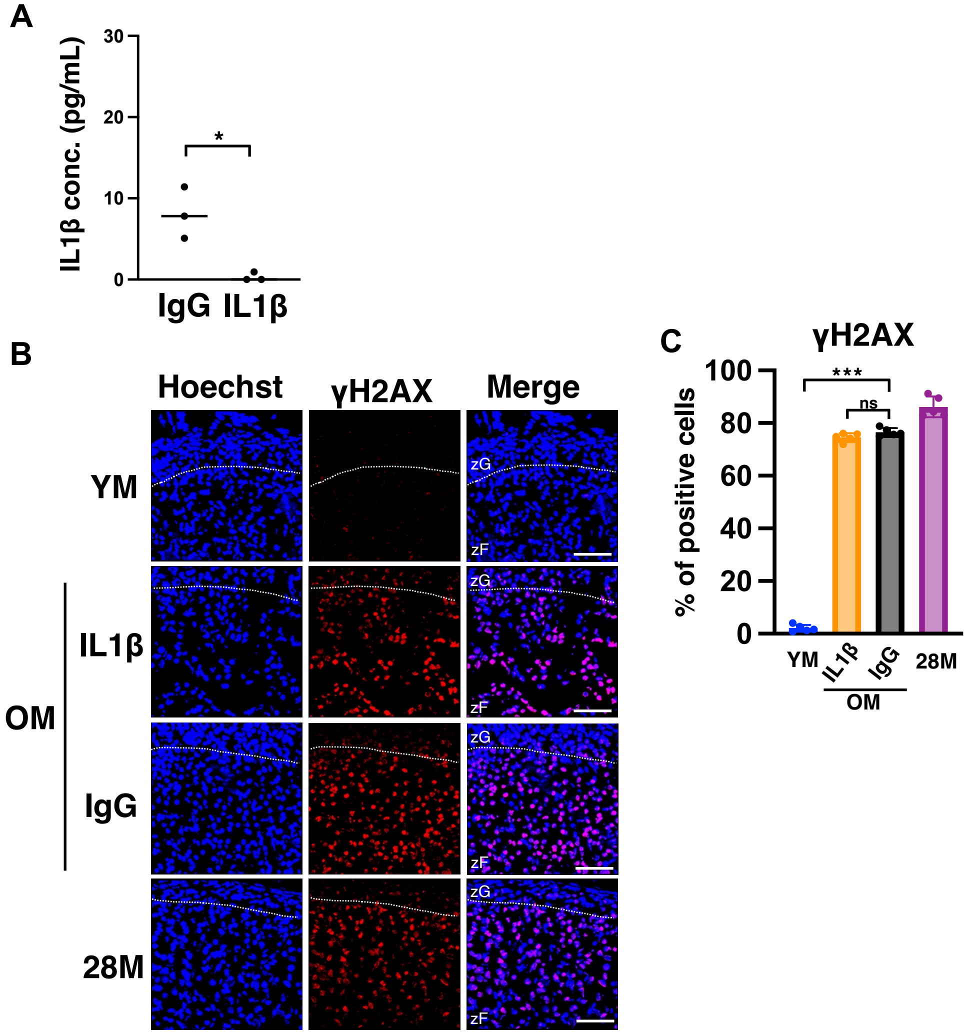

Figure S6. A. Measurements of IL1β levels in mouse plasma in treated with IgG and IL1β-antibody mice (IgG: control IgG, IL1β: IL1β-antibody). **B**. Immunofluorescence analysis of γH2AX in the adrenal gland of IL1β-antibody-treated mice. Each section was stained with anti-γH2AX antibodies and observed using confocal laser scanning microscopy. Scale bar = 50 μm. zG: zona glomerulosa, zF: zona fasciculata. (YM: young mice, IL1β and IgG: antibody administered mice, 28M: 28-month-old mice). **C**. Percentage of γH2AX-positive cells. Data are shown as mean ± SD. Asterisks indicate statistical significance (**P* < 0.05, ****P* < 0.0001, ns; not significant). The quantitative results of immunostaining are summarized in Table S1.

Supplementary Figure 7

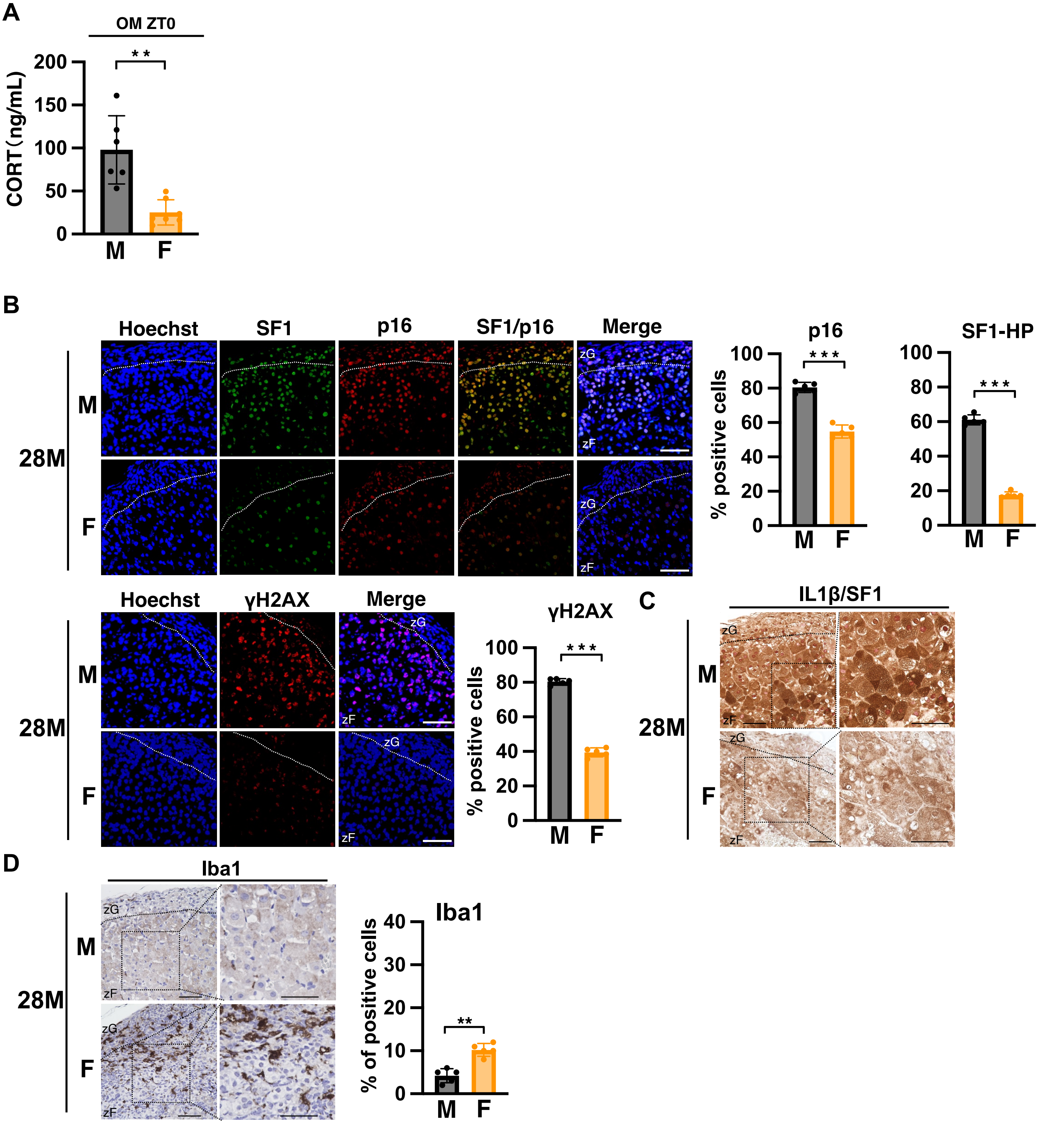

Figure S7. Comparison of CORT levels at ZT0 and markers of senescence in males and females. A. Comparison of CORT levels at ZT0 in mouse plasma samples measured in >26-month-old male and female mice. **B–D.** Immunofluorescence analysis of p16, SF1, γH2AX, IL1β and Iba1 in the adrenal gland. Each section was stained with anti-p16, -SF1, -γH2AX, -IL1β and -Iba1 antibodies and observed using confocal laser scanning microscopy and light microscopy. M: male; F: female; 28M: 28-month-old mice. C: IL1β and SF1 in brown and red, respectively. D: Iba1 and nuclear in brown and blue, respectively. Scale bar (solid line): 50 μm. zG: zona glomerulosa; zF: zona fasciculata. Data are presented as means ± SDs. Asterisks indicate statistical significance (***P* < 0.001, and ****P* < 0.0001; ns: not significant). Quantitative results of immunostaining are summarized in Table S1.

Supplementary Figure 8

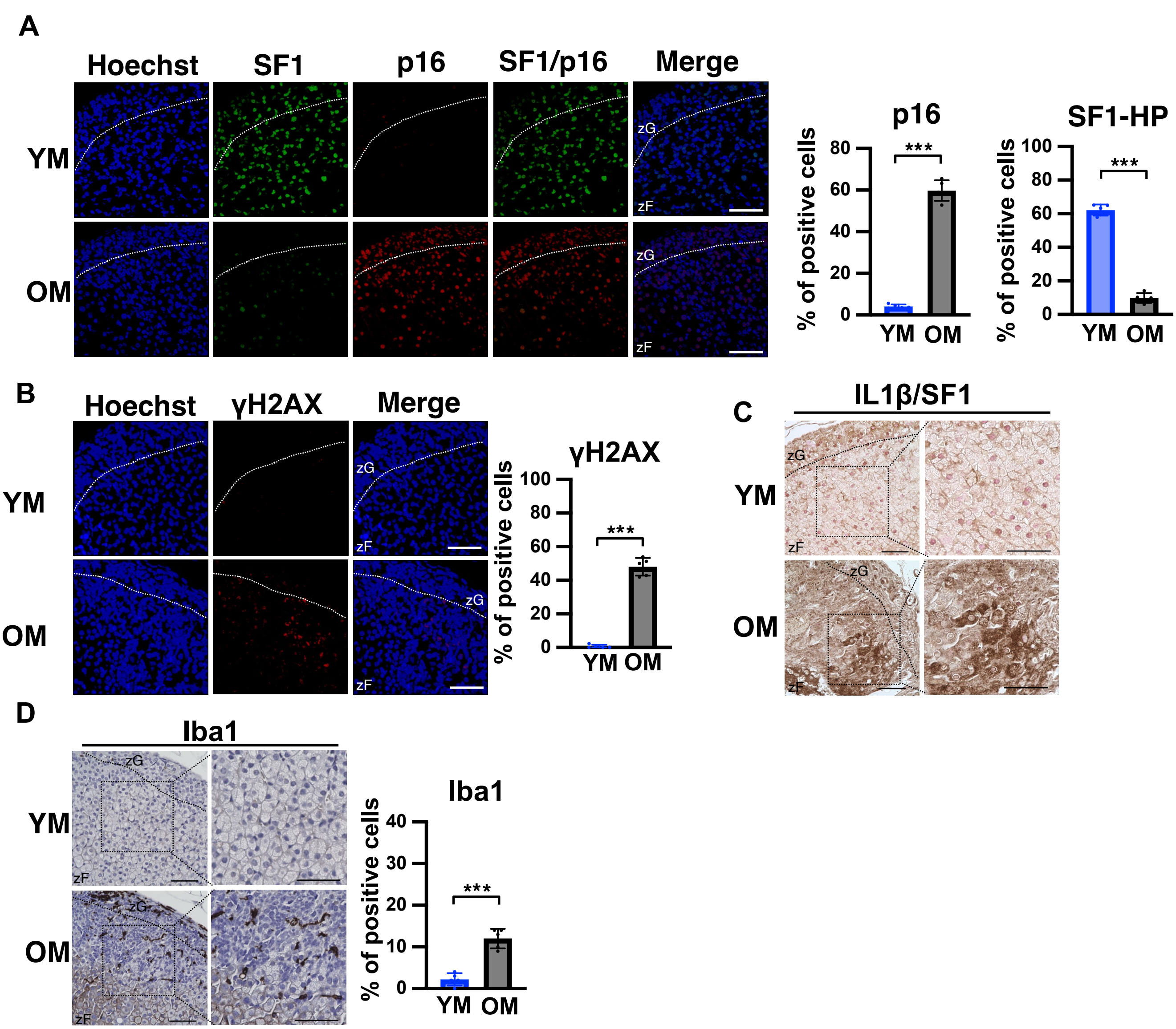

Figure S8. Adrenal glands in old female mice have increased markers of senescence. A–D. Immunofluorescence analysis of p16, SF1, γH2AX, IL1β, Iba1 in the adrenal gland in YM and OM at ZT0, respectively, using confocal laser scanning microscopy and light microscopy. C; IL1β (brown), SF1(red). D; Iba1(brown), nuclear (blue). Scale bar (solid line) = 50 μm. zG: zona glomerulosa, zF: zona fasciculata. Percentage of positive cells was quantified in YM and OM at ZT0 in zF. Data are shown as mean ± SD. Asterisks indicate statistical significance (****P* < 0.0001, ns; not significant). The quantitative results of immunostaining are summarized in Table S1.

Supplementary Figure 9

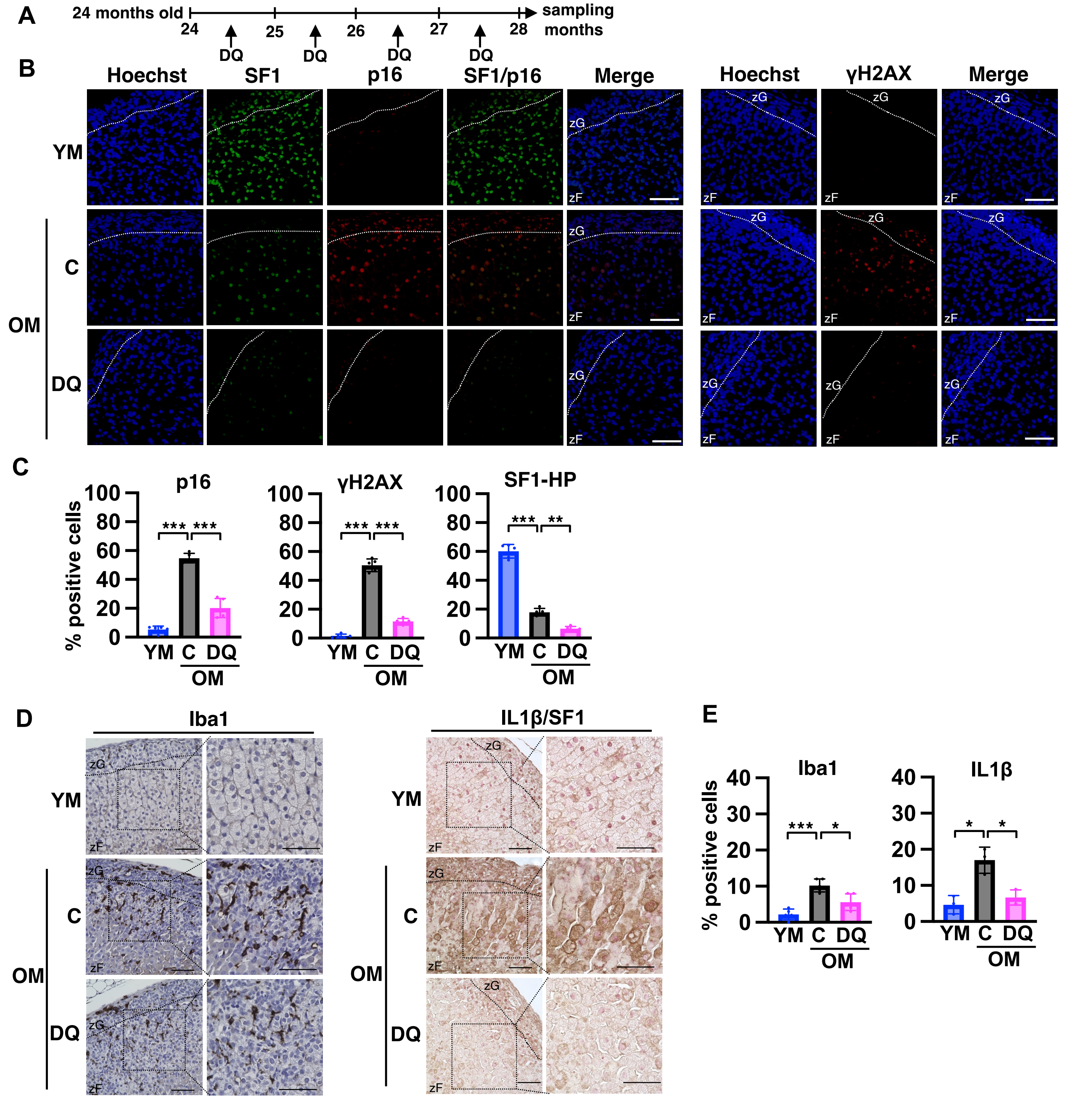

Figure S9. Senescence markers in old female mice are recovered by senolytic drugs. A. Schedule of DQ administration experiments. **B and D.** Immunofluorescence analysis of p16, γH2AX, SF1, Iba1 and IL1β in the adrenal gland of DQ-treated mice. Each section was stained with anti-p16, -γH2AX, -SF1, -Iba1 and -IL1β antibodies and observed using confocal laser scanning microscopy and light microscopy. Scale bar (solid line) = 50 μm. zG: zona glomerulosa, zF: zona fasciculata. (C: control, DQ: senolysis). **C and E.** The number of p16-, γH2AX, SF1, Iba1 and IL1β-positive cells in zF was quantified. D; Iba1 and IL1β (brown), nuclear (blue) and SF1 (red). SF1-HP; SF1-positive cells. (**P < 0.001, ***P < 0.0001, ns; not significant). The quantitative results of immunostaining are summarized in Table S1.

Supplementary Figure 10

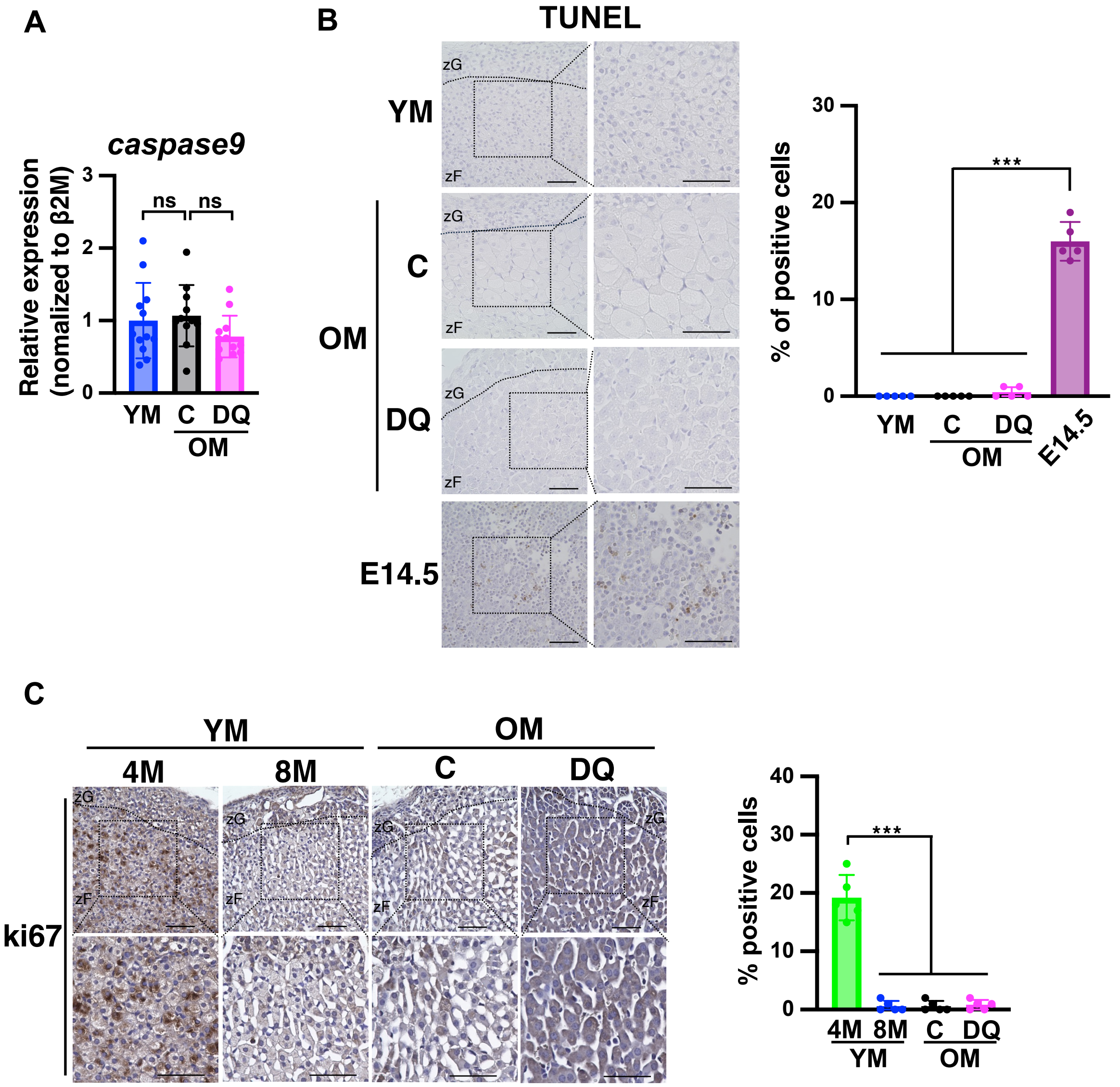

Figure S10. A. Analysis of mRNA expression of *caspase9* in YM- and OM-DQ-treated mice. All data are presented as relative values and normalized to β-2 microglobulin. **B and C.** Immunofluorescence analysis of TUNEL and ki67 staining in the adrenal gland of DQ-treated mice. Each section was observed using a light microscope. TUNEL (B) and ki67 (C); brown, nuclear; blue. Scale bar (solid line) = 50 μm. zG: zona glomerulosa, zF: zona fasciculata. (YM: young mice, C: control, DQ: senolysis, E14.5: embryonic 14.5-day-old mice, 4M: 4-month-old mice, 8M: 8-month-old mice). Percentage of positive cells for TUNEL and ki67 staining. Data are shown as the mean ± SD. Asterisks indicate statistical significance (***P < 0.0001, ns; not significant). The quantitative results of immunostaining are summarized in Table S1.

Supplementary Figure 11

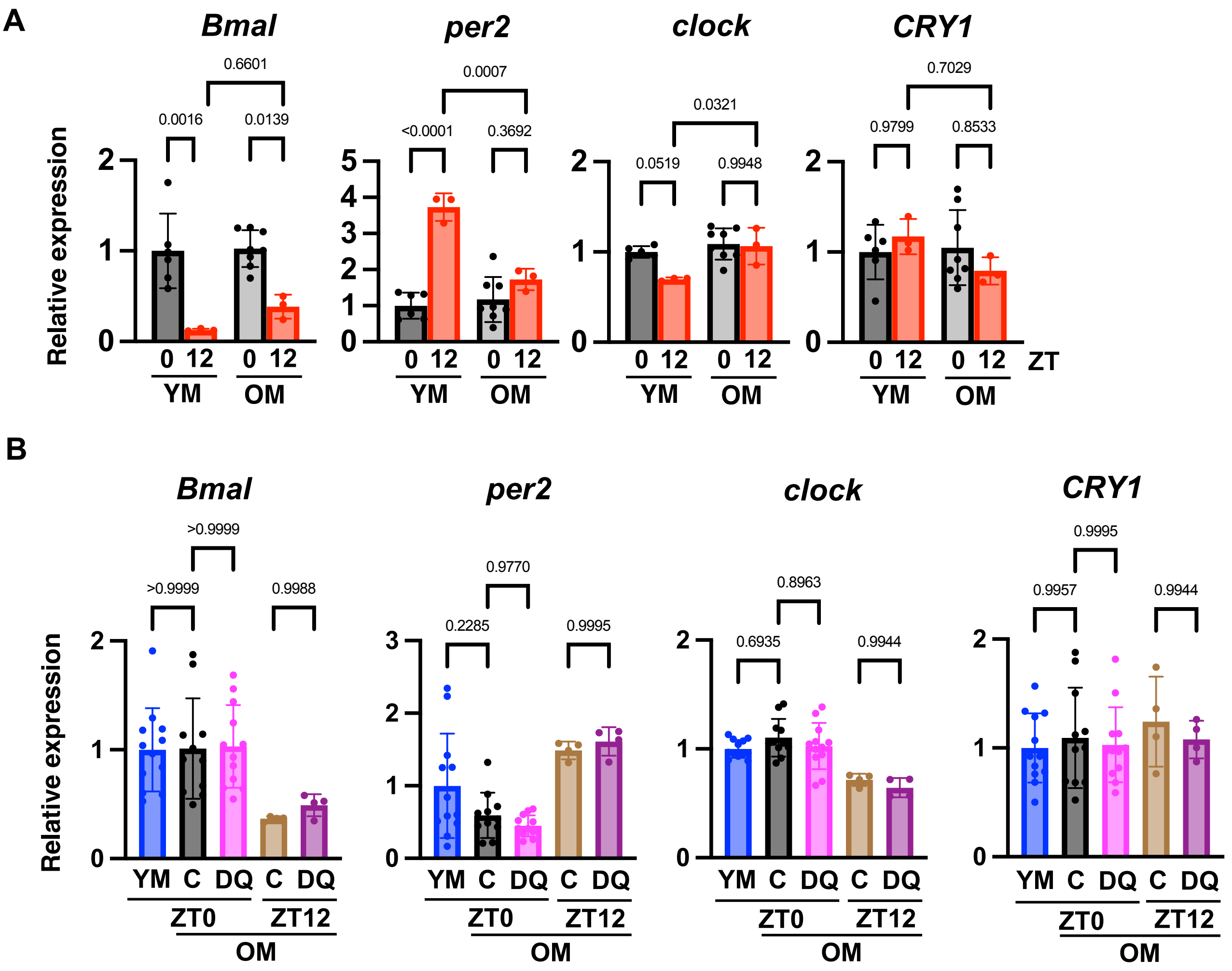

Figure S11. A and B. Analysis of mRNA expression of clock genes in YM and OM at ZT0 and ZT12 and YM- and DQ-treated mice. All data are presented as relative values and normalized to TBP. Data are shown as the mean ± SD.
