# Supplementary material for: Accumulation of senescent cells in the adrenal gland induces hypersecretion of corticosterone via IL1β secretion
Source: Aging Cell. 2024 May 20;23(9):e14206. doi: 10.1111/acel.14206 (PMC11488315; doi:10.1111/acel.14206)
Supplement: Supplementary file 2 — Tables S1–S3 [file ACEL-23-e14206-s001.pdf]

**Supplementary Table 1**. List of Quantitative Results of Immunostaining.

| Figure 1 G-I | SF1-HP | StAR | SF1-HP/StAR |
|---|---|---|---|
| YM ZT0 | 7.4±1.1 | 4.5±1.9 | 3.1±0.9 |
| YM ZT12 | 7.5±1.5 | 13.22±2.1 | 5.6±0.5 |
| OM ZT0 | 59.3±4.0 | 76.6±3.5 | 47.5±2.3 |
| OM ZT12 | 61.7±1.8 | 77.6±3.2 | 49.9±2.7 |

| Figure 2 E | p16 | γH2AX |
|---|---|---|
| 6M | 5.0±2.6 | 9.0±1.8 |
| 18M | 55.0±3.2 | 42.3±5.4 |
| 24M | 84.1±5.1 | 75.5±4.9 |

| Figure 2G | 53BP1 | βgal |
|---|---|---|
| YM | 5.0±2.5 | 1.8±0.76 |
| OM | 67.7±5.2 | 52.0±3.3 |

| Figure 2 I | p16 | SF1-HP |
|---|---|---|
| YM ZT0 | 4.0±1.3 | 6.3±2.5 |
| YM ZT12 | 5.7±1.8 | 13.6±2.4 |
| OM ZT0 | 83.4±2.1 | 56.0±3.7 |
| OM ZT12 | 82.7±2.2 | 58.4±3.0 |

| Figure 3 G, H, J | p16 | SF1-HP | StAR |
|---|---|---|---|
| Control (OM) | 82.1±4.5 | 57.1±3.7 | 71.3±5.3 |
| DQ (OM) | 19.9±9.0 | 20.1±4.9 | 2.5±2.4 |

| Figure 4 C | SF1-HP |
|---|---|
| OM IL1β (+) | 70.9±12.0 |
| OM IL1β (-) | 72.4±15.4 |

| Figure 4 C | IL1β |
|---|---|
| YM | 4.6±2.8 |
| OM | 50.0±21.9 |

| Figure 4 E | IL1β |
|---|---|
| Control (OM) | 47.7±21.1 |
| DQ (OM) | 9.2±2.8 |

| Figure 4 I | Iba1 |
|---|---|
| YM | 3.8±1.3 |
| Control (OM) | 4.4±1.5 |
| DQ (OM) | 6.0±1.6 |

| Figure 5 E, F, G | p16 | SF1-HP | StAR |
|---|---|---|---|
| YM | 1.6±1.6 | 3.8±1.0 | 11.6±2.2 |
| α-IL1β OM | 30.7±2.0 | 9.4±1.7 | 30.7±2.0 |
| α-IgG OM | 63.1±5.2 | 48.2±4.6 | 72.0±3.8 |
| 28M | 75.1±4.2 | 66.1±2.6 | 71.5±5.7 |

| Figure 5 I, K, L | Iba1 | IL1β | SF1-HP |
|---|---|---|---|
| α-IL1β OM | 5.2±1.6 | 20.7±6.0 | 10.4±5.7 |
| α-IgG OM | 5.0±2.0 | 48.7±19.0 | 62.7±2.5 |

| Figure 5 M | SF1-HP |
|---|---|
| α-IL1β OM IL1β (-) | 5.3±1.5 |
| α-IgG OM IL1β (-) | 58.0±1.0 |
| α-IL1β OM IL1β (+) | 17.0±7.6 |
| α-IgG OM IL1β (+) | 65.7±2.3 |

| Figure 6 C, D, F | p16 | γH2AX | Iba1 |
|---|---|---|---|
| Control | 10.7±1.7 | 0.0±0.0 | 3.2±1.3 |
| Control Dex | 50.6±2.6 | 72.1±5.0 | 3.8±1.8 |
| DQ Dex | 6.7±1.8 | 29.0±4.1 | 4.0±1.6 |
| OM | 75.9±5.5 | 81.6±2.0 | 3.8±1.9 |

| Figure S4 D, E | p16 | SF1-HP |
|---|---|---|
| 6M | 2.2±1.5 | 8.6±1.9 |
| 18M | 66.6±7.2 | 29.3±4.7 |
| 24M | 89.8±3.9 | 56.7±2.2 |

| Figure S5 A | p16 | γH2AX | βgal |
|---|---|---|---|
| Control (OM) | 46.6±4.3 | 72.8±9.6 | 39.2±9.2 |
| DQ (OM) | 8.0±2.4 | 8.6±1.6 | 1.6±2.0 |

| Figure S5 B | p16 | γH2AX | 53BP1 | βgal |
|---|---|---|---|---|
| Control (OM) | 83.2±3.3 | 65.4±18.3 | 67.5±2.7 | 68.9±15.0 |
| DQ (OM) | 26.4±5.5 | 28.9±4.9 | 22.1±1.9 | 23.3±4.9 |

| Figure S6 C | γH2AX |
|---|---|
| YM | 2.1±1.3 |
| α-IL1β OM | 74.6±1.6 |
| α-IgG OM | 76.6±1.6 |
| 28M | 86.2±3.9 |

| Figure S7 B, D | p16 | SF1-HP | γH2AX | Iba1 |
|---|---|---|---|---|
| 28M Male | 80.4±3.0 | 61.1±2.9 | 80.4±1.8 | 4.2±1.6 |
| 28M Female | 54.8±3.8 | 17.6±1.8 | 39.5±2.6 | 10.2±1.5 |

| Figure S8 A, B, D | p16 | SF1-HP | γH2AX | Iba1 |
|---|---|---|---|---|
| YM | 4.1±1.0 | 62.2±3.3 | 0.6±1.0 | 2.2±1.5 |
| OM | 59.8±4.9 | 9.8±2.9 | 48.0±5.3 | 12.0±2.3 |

| Figure S9 C, E | p16 | γH2AX | SF1-HP | Iba1 | IL1β |
|---|---|---|---|---|---|
| YM | 5.3±2.3 | 1.4±1.4 | 60.2±4.8 | 2.2±1.5 | 4.7±2.5 |
| Control (OM) | 54.8±3.2 | 50.5±4.4 | 17.9±2.6 | 10.2±1.8 | 17.0±3.6 |
| DQ (OM) | 20.2±6.6 | 11.7±2.0 | 6.6±1.4 | 5.6±2.3 | 6.7±2.1 |

| Figure S10 B | TUNEL |
|---|---|
| YM | 0.0±0.0 |
| Control (OM) | 0.0±0.0 |
| DQ (OM) | 0.4±0.5 |
| E14.5 | 16.0±2.0 |

| Figure S10 C | Ki67 |
|---|---|
| 4M (YM) | 19.2±3.9 |
| 8M (YM) | 0.6±0.9 |
| Control (OM) | 0.6±0.9 |
| DQ (OM) | 0.8±0.8 |

**Supplementary Table 2** The primer sequences used in this study are listed.

| primer name | Sequence (5' to 3') |
|---|---|
| mop16-Fw | CCAGGGCCGTGTGCAT |
| mop16-Rv | TACGTGAACGTTGCCCATCA |
| moSF1-Fw | AGTTGCTATTGTGCCTGGTG |
| moSF1-Rv | TCGTTGCCCAAATGCTTGTG |
| moStAR-Fw | TTGGGCATACTCAACAACCA |
| moStAR-Rv | TGATGACCGTGTCTTTTCCA |
| moCYP11A1-Fw | GCTGGAAGGTGTAGCTCAGG |
| moCYP11A1-Rv | CACTGGTGTGGAACATCTGG |
| moCYP11B1-Fw | CAGGAGCCTGACCCGATGGACA |
| moCYP11B1-Rv | GACTGTGGACGACCCTCTGCCA |
| moPer2-Fw | ACGACAATGGGAAGGAGCTG |
| moPer2-Rv | TGTGCTCTGCCTCTGTCATC |
| moBmal-Fw | CAAGCACCTTCCTTCCAATG |
| moBmal-RV | GATTGCAGTCCACACCACTG |
| moclock-Fw | CTCCACATGCCTCCCACTTT |
| moclock-Rv | ATCCCCAGGCATGAGAGTCT |
| moCry1-Fw | GGTTGCCTGTTTCCTGACTCGT |
| moCry1-Rv | GACAGCCACATCCAACTTCCAG |
| moTBP-Fw | TCAAACCCAGAATTGTTCTCC |
| moTBP-Rv | GGGGTAGATGTTTTCAAATGC |
| moMRC1-Fw | CACACTCATCCATTACAACCAAA |
| moMRC1-Rv | GAGGACCACGGTGACCACT |
| moCD68-Fw | TCTCTAAGGCTACAGGCTGCT |
| moCD68-Rv | CAATGATGAGAGGCAGCAAG |
| moTREM2-Fw | GACTGTGGCCAAGATGCTG |
| moTREM2-Rv | CCTGGCTGGACTTAAGCTGT |
| moCSF1-Fw | AAGTGGAGGAGCCATCGAG |
| moCSF1-Rv | ACTGGCAGTTCCACCTGTCT |
| moF4/80-Fw | CGTGTTGTTGGTGGCACTGTGA |
| moF4/80-Rv | CCACATCAGTGTTCCAGGAGAC |
| moIL1b-Fw | TGGACCTTCCAGGATGAGGACA |
| moIL1b-Rv | GTTCATCTCGGAGCCTGTAGTG |
| moCCL2-Fw | GCTACAAGAGGATCACCAGCAG |
| moCCL2-Rv | GTCTGGACCCATTCCTTCTTGG |
| moCD11b-Fw | TACTTCGGGCAGTCTCTGAGTG |
| moCD11b-Rv | ATGGTTGCCTCCAGTCTCAGCA |
| moCX3CL1-Fw | TGCTCATCCGCTATCAGCTA |
| moCX3CL1-Rv | ATTTCTCCTTCGGGTCAGC |
| moCXCL1-Fw | TCCAGAGCTTGAAGGTGTTGCC |
| moCXCL1-Rv | AACCAAGGGAGCTTCAGGGTCA |
| moIL6-Fw | TACCACTTCACAAGTCGGAGGC |
| moIL6-Rv | CTGCAAGTGCATCATCGTTGTTC |
| moCCL5-Fw | CCTGCTGCTTTGCCTACCTCTC |
| moCCL5-Rv | ACACACTTGGCGGTTCCTTCGA |
| moMMP3-Fw | CTCTGGAACCTGAGACATCACC |
| moMMP3-Rv | AGGAGTCCTGAGAGATTTGCGC |
| moTNFα-Fw | GGTGCCTATGTCTCAGCCTCTT |
| moTNFα-Rv | GCCATAGAACTGATGAGAGGGAG |
| moCasapase9-Fw | AGAGGTTCTCAGACCAGAAACA |
| moCasapase9-Rv | CATATCTGCATGTCCCCTGA |
| moMicroglobulin-Fw | CCACTGAAAAAGATGAGTATGCCT |
| moMicroglobulin-Rv | CCAATCCAAATGCGGCATCTTCA |

**Supplementary Table 3** The manufacturers and conditions of use of the antibodies used in this study are summarized below.

| Antibody name | Supplier (Catalog No.) | Clonality (Clone) | Reactivity | Application |
|---|---|---|---|---|
| Anti-CDKN2A/p16INK4a Antibody | abcam (ab211542) | Rabbit mono (EPR20418) | Mouse | IF (1:200) |
| Anti-mouse Nr5a1 (Ad4BP/SF1) monoclonal Antibody rat mAb | Transgenic (KO610) | mouse mono (1B1F10) | Mouse | IF (1:500)<br>IHC (1:200) |
| StAR Rabbit Polyclonal antibody | Proteintech (12225-1-AP) | Rabbit poly | Human<br>Mouse<br>Rat<br>Pig | IF (1:100) |
| Phospho-Histone H2A.X(Ser139) (20E3) Rabbit mAb | Cell Signaling Technology (#9718) | Rabbit mono (20E3) | Human<br>Mouse<br>Others | IF (1:500) |
| 53BP1 antibody | Novusbio (NB100-304) | Rabbit poly | Human<br>Mouse<br>Others | IF (1:250) |
| ki67 antibody | Novusbio (NB110-89719) | Rabbit poly | Human<br>Mouse<br>Rat | IHC (1:100) |
| Iba1 antibody | GeneTex (GTX100042) | Rabbit poly | Human<br>Mouse<br>Rat | IHC (1:50) |
| IL1β antibody | Bioss (bs-0812R) | Rabbit poly | Human<br>Mouse<br>Others | IHC (1:100) |
| Cy3-conjugated Donkey Anti-Rat IgG (H+L) | Jackson Immuno Research (712-165-153) | - | - | IF secondary antibody (1:1000) |
| Alexa Fluor 647 Goat anti-rabbit IgG (H+L) | Thermo Fisher Scientific (A-21245) | - | - | IF secondary antibody (1:1000) |
